# Thermal efficiency dataset around Cuban seas (TEDACS)

**DOI:** 10.12688/openreseurope.16815.1

**Published:** 2024-01-10

**Authors:** Alejandro Rodriguez, Melissa Abreu, Dailin Reyes, Melany Abreu, Humberto L. Varona, Carlos Noriega, Amilcar Calzada, Moacyr Araujo

**Affiliations:** 1 Marine Meteorology Center (CMM), National Institute of Meteorology (INSMET)., Havana, Havana, 11700, Cuba; 2 Laboratory of Physical, Coastal and Estuarine Oceanography (LOFEC)., Federal University of Pernambuco, Pernambuco, Recife-PE, Brazil; 3 Department of Oceanography (DOCEAN)., Federal University of Pernambuco. Recife-PE. Brazil., Pernambuco, Recife-PE, Brazil; 4 Center for Risk Analysis, Reliability Engineering and Environmental Modeling (CEERMA)., Federal University of Pernambuco. Recife-PE. Brazil., Pernambuco, Recife-PE, Brazil; 5 Brazilian Research Network on Global Climate Change (Rede CLIMA)., São José dos Campos, São Paulo, Brazil

**Keywords:** Thermal efficiency, OTEC technologies, Sea thermal energy, Sea Surface Temperature

## Abstract

Currently, the generation of electrical energy in Cuba is supported by oil and natural gas. These sources, as it is known, are directly linked to large emissions of pollutants that are released into the environment. Therefore, it is necessary to search for new energy options that are directed towards sustainable development, allowing the preservation of natural ecosystems. Owing to the location and geographical characteristics of Cuba, it is necessary to assess the energy possibilities of the seas that surround it and to search for the most feasible areas to obtain energy from the sea temperature. This renewable energy source, in addition to being used to generate electricity, can also be used in derived technologies, such as desalination, refrigeration, and aquaculture.

Hence, a dataset is presented with the calculation of the thermal efficiency for the exploitation of thermal energy from the sea, which is based on the thermal gradient between the sea potential temperatures between the shore and the level of depth being analyzed. Outputs of 27 years of daily data from the Copernicus Marine Environmental Monitoring Service (CMEMS) GLOBAL_MULTIYEAR_PHY_001_030 product with a spatial resolution of 1/12° were used. The calculation was made using a Python script of the daily thermal efficiency at depths of 763, 902, and 1062 m, as these are the levels that are traditionally studied for the exploitation of sea thermal energy. In this way, 27 files of each level were generated for a total of 81 files in text format separated by commas. Each file is presented with the date, level, coordinates, and thermal efficiency. The dataset is available from the Science Data Bank repository ( https://doi.org/10.57760/sciencedb.10037).

## Introduction

Ocean thermal energy conversion (OTEC) is a renewable energy technology that harnesses the solar energy absorbed by oceans to generate electricity. Heat from the sun warms surface water more than deep ocean water, creating a naturally available temperature gradient in the ocean. OTEC uses warm sea surface water with a temperature of approximately 25°C to vaporize a working fluid, which has a low boiling point, such as ammonia and propane. The vapor expands and turns a turbine coupled with a generator to produce electricity ^1^. The steam was then cooled with seawater that has been pumped up from the deepest layer of the ocean, where the temperature was approximately 5°C.

Thermal efficiency is the amount of heat/power used in a Rankine cycle for converting oceanic thermal energy into electrical energy ^2^. Among the different approaches used for the analysis of thermal energy efficiency, an approach based on a theoretical limit is used to maximize the efficiency of an OTEC system by converting the heat stored in the warm surface waters of the oceans into mechanical work ^1^.

According to previous studies, the temperature of the sea surface around Cuba oscillates throughout the year between 26° and 30°, on average. These characteristics indicate the convenience of studying the thermal energy of the seas around Cuba ^3^.

The present work exposes the main features of a dataset that contains the computation of thermal efficiency in the seas surrounding Cuba between latitudes 18.5° and 24.0° and longitudes -73.5° and -85.5°. The objective is to evaluate the study area according to the requirements for obtaining thermal energy from the sea, which must reach values greater than or equal to 0.7 of thermal efficiency ^4^. For the computation, 27 years of global ocean reanalysis data from the GLOBAL_MULTIYEAR_PHY_001_030 product of the Copernicus Marine Environmental Monitoring Service (CMEMS) with a spatial resolution of 1/12° were used ^5^.

With this dataset presented, it is also possible to develop other investigations in the study area to advance the knowledge of the thermal energy potential of the sea to investigate the selection of sites where OTEC plants can be built for the generation of electrical energy, desalination seawater, and other applications.

In the following sections, the methods are presented, which introduce the study area, equation for calculating thermal efficiency, dataset with its structure, generated files, and availability of data.

## Methods

This study was performed using the output cmems_mod_glo_phy_my_0.083_P1D-m, available for free download from the Copernicus Marine Environment Monitoring Service (CMEMS) GLOBAL_MULTIYEAR_PHY_001_030 ( https://data.marine.copernicus.eu/product/GLOBAL_MULTIYEAR_PHY_001_030/services), that simulates the global ocean at 1/12° (approximately 8 km) resolution ^5^, using the space-time evolution of the 3D thermodynamic variables temperature and salinity (T, S) at 4 levels: -0.49, -763.33, -902.34 and -1062.44 m. Were downloaded 27 years of these files with the "motuclient-python" tool ^6^, which were then manipulated and analyzed with the CDO **adisit** function ^7^, to calculate the temperature in situ from potential temperature and salinity data. The calculations of the maximum efficiency and the generation of the maximum, annual, and seasonal efficiency maps for each level were performed using Python version 3.9 (RRID:SCR_008394) of Python language. Therefore, the presented dataset contains the computation of thermal efficiency in the seas surrounding Cuba between latitudes 18.5° and 24.0° and longitudes -73.5° and -85.5° ^4^.

## Brief characterization of the study area

The study area is influenced by the marine current systems that surround it: The Caribbean current, which extends from the arc of the Lesser Antilles to the vicinity of the Yucatan Peninsula; the Yucatan Current, which connects the Caribbean Sea and the Gulf of Mexico; the Loop Current, a flow that joins the Yucatan current and the La Florida current in the eastern part of the Gulf of Mexico; and the Florida Current, from the Straits of Florida to Cape Hatteras, and is considered the beginning of the Gulf Stream. Trend studies have observed that marine flow in the Caribbean Sea has had a slight decrease in magnitude, while in the Gulf of Mexico and the Near Atlantic, there has been a subtle increase ^8^.

**Figure 1.**
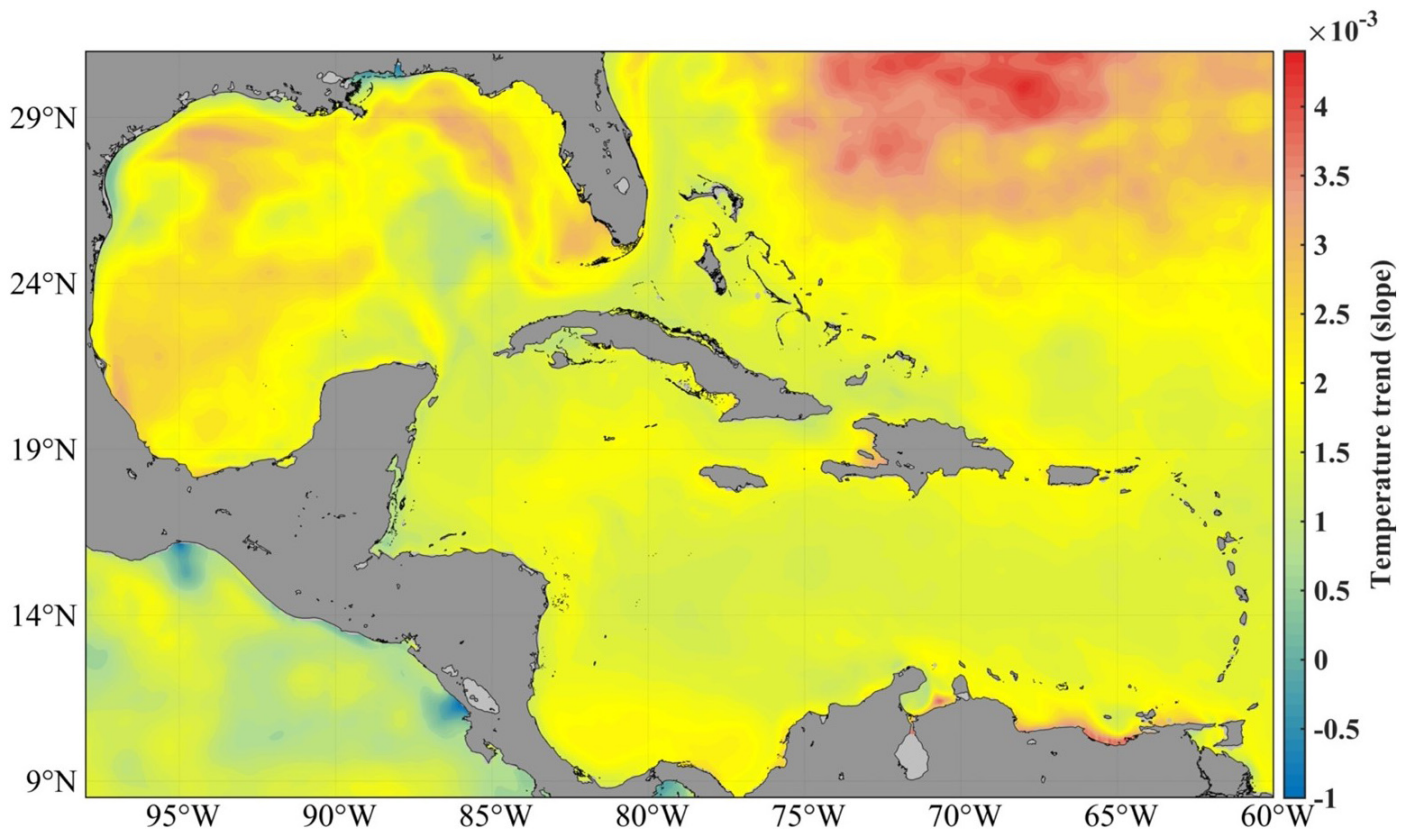
SST trend from GLOBAL_MULTIYEAR_PHY_001_030 product, 1993–2018 ^5^.

In this area, the average temperature of the sea surface oscillates between 25 and 30º, with a minimum in February and a maximum in September. The maxima are found over deep waters in the area of the Casilda-Cazones Gulf and south of Isla de la Juventud, towards the central and northern Caribbean area, as well as another maximum north of the western coasts. Extreme minimum values were recorded in the Gulf of Mexico area, with 24° and maximums values of up to 31°. Calculation of the sea surface temperature trend with the reanalysis data used in this research revealed an increase between 0.5° and 2.5° ( Figure 1).

**Figure 2.**
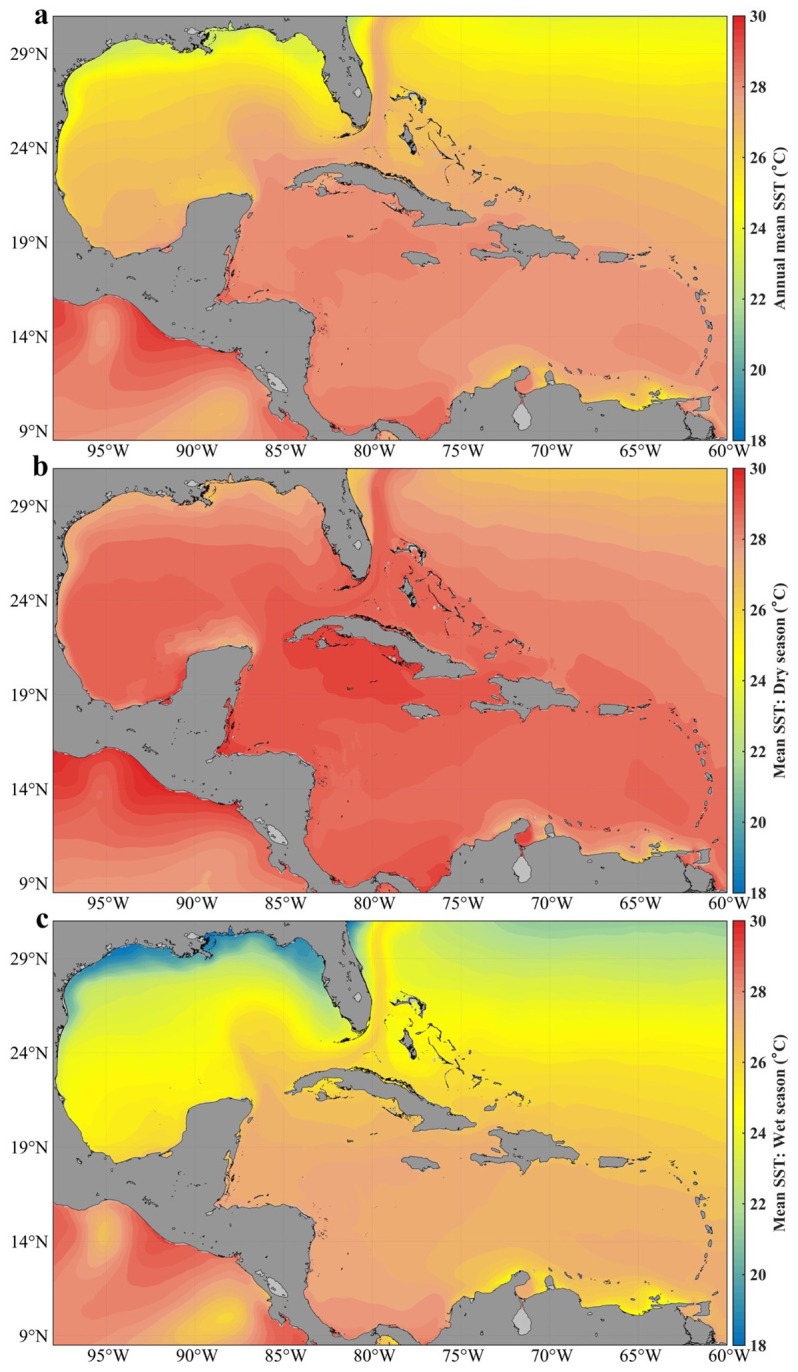
Analysis of the mean sea surface temperature in Cuban waters with the GLOBAL_MULTIYEAR_PHY_001_030 product in the Caribbean Sea and the Gulf of Mexico ^5^: **a**) distribution of the annual mean, **b**) distribution in the dry season, **c**) distribution in the wet season.

The area is also affected by teleconnection events such as the NAO and ENSO, showing a zonal pattern in the correlation between the sea surface temperature and the NAO index six months before the development of the negative and positive phases of the event ^9^. In addition, extreme phenomena such as hurricanes and cold fronts affect the Cuban archipelago every year, highlighting the relationship between the intensity of these phenomena and the different phases of the NAO and ENSO ^10^. Figure 2 a shows the annual spatial distribution of sea surface temperature (SST) in Cuban waters, while Figure 2 b and c show the seasonal spatial distribution.

## Bathymetric characterization

**Figure 3.**
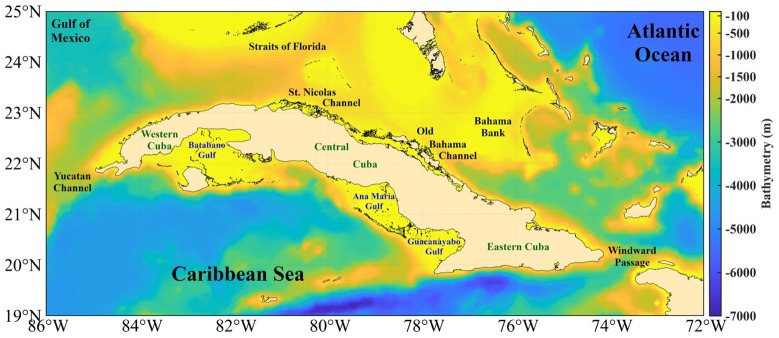
Bathymetry of the seawaters around Cuba taken from the GEBCO Bathymetric Compilation Group ^13^ under GEBCO 2018 Licence.

Cuba is geographically located in the American Mediterranean, between longitudes 74°7'52" W and 84°54'57" W, and latitudes 19°49'36" N and 23°17'9" N ( Figure 3). It is bordered to the north by the Gulf of Mexico, Florida Strait, St. Nicholas Channel, and old Bahama Channel; to the south by the western Caribbean Sea and Strait of Columbus; to the west by the Yucatan Channel; and to the east by the Windward Passage ^11^. The westernmost and northernmost island platforms contain the Guanahacabibes Gulf, with depths of 5–25m. Less than 1 mile from the outer edge, the shelf's insular slope drops steeply from 10 to 100m. Reefs appear parallel to the coast, cut by numerous ravines, and pass between the 5 and 10m isobaths, which form a chain that obstructs access to the coast. Continuing along the western north of the Cuban archipelago, the insular slope presents an abrupt, steep, and sinuous drop, very close to the coast, which causes the 200m isobath to be frequently found less than 1 mile from the slope.

The island platform, in the northern central zone (from Punta Hicacos to Punta Maternillos), is wide, and its edge generally registers depths between 10–25m; outside the edge of the platform, the depths increase sharply, so the island slope varies from to 20–200m depth in less than 1m of distance. Above this island platform, the most continuous and extensive coral barrier in Cuba extends, with depths of less than 10m and in many places less than 5m, with cays and heads that emerge. In the northeastern area (from Nuevitas Bay to Punta Maisí), the island platform, less than 10m in depth, has an edge of 3 miles from the coast. Outside the island shore, there are no shallows, heads, or reefs that are dangerous to navigation, and the depths increase abruptly; the 200m isobath crosses within one mile of the border throughout the entire region ^12^.

Continuing along the southeastern area, the entire coast is bordered by a low strait of less than 20m depth, whose edge corresponds to the vertical slope that the Eastern Trench presents in this part of the coast. The 200m isobath crosses very close to the coast, over an underwater relief that reaches great depths to the south, so the island platform of the Island of Cuba becomes very narrow, and the slope falls abruptly towards abyssal depths where it is located. The Eastern trench reaches 7,239m depth. Further to the west south, in the approaches to the Guacanayabo Gulf from the sea, the depths are great and the 200m isobath crosses 1.6 miles southwest of Cay Cruz. The isobath is the edge of the island platform, made up of coral reefs submerged at a depth of 20–40m. Among these reefs, depths are generally less than 20m, with a gentle slope ^12^.

Continuing westwards to the south, the Ana María Gulf is separated from the Guacanayabo Gulf by the cays Mate and Laberinto de las Doce Leguas ( Figure 3); and to the southwest it is limited by the barrier reef of the Jardines de la Reina Archipelago, which extends 118 miles to the northwest with depths mostly less than 5m. The greatest distance between the edge of the platform and the coral reef barrier does not exceed 2 miles. Beyond this, the depths are abyssal, located at distances between 16 and 20 miles, with depths of 22–58m. From the Punta María Aguilar to the Cazones Gulf, the shallow area at less than 10m depth that borders the coast is very narrow, and there are places where the cliffed coast falls directly to greater depths. Depths of 100m were recorded 2 miles from the coast and 2,000m in the central part of the Gulf of Cazones.

Next, from south to west, the underwater relief of the Batabanó Gulf has some irregularities due to the existence of cays and shoals with depths between 2–5m ( Figure 3). In the central part, the underwater relief of the gulf is gentle, with maximum depths between 6–7m, and a shallow slope. In the westernmost area of the southern portion, from Guano del Este Cay to San Antonio Cape, the slope of the island platform of Cuba falls abruptly towards great depths, finding in many places an isobath of 1,000m very close to the edge of the platform. Finally, the southern and southeastern coasts of Isla de la Juventud, with steep cliffs, drop abruptly to abyssal depths of 3,000m ^12^.

## Computation of thermal efficiency

In the most feasible marine areas for the operation of OTEC plants, the average surface temperature of each year is approximately 26.7 to 29.4°, with cold water available at 4.4° or less at a depth of 900–1000m ^14^. Therefore, even without the inevitable reduction caused by friction and heat loss, the maximum efficiency achieved by heat conversion in an OTEC plant can be achieved with a very small rate of energy production. To apply the calculation of thermal efficiency, the approach proposed in the research carried out in was considered, which is based on a theoretical limit, up to a maximum efficiency of an OTEC system through the conversion of heat into mechanical work stored in the warm surface waters of the tropical oceans:


$$\eta \;=\;\frac{Tw-Tc}{Tw}$$

Where η: thermal efficiency.

Tw: hot water temperature.

Tc: cold water temperature.

The calculation was made with a fixed longitude: moving in latitude to the north from the north coast of the archipelago and moving in latitude to the south from the south coast ^15^.

### Data validation

The MODIS Aqua Level 3 dataset encompasses sea surface temperature (SST) information derived from the NASA MODIS sensor situated on the Aqua satellite. SST data were extracted from the thermal infrared spectrum with wavelengths of 11 and 12 μm. After processing, the data is projected onto a cylindrical equidistant map with spatial bins measuring either 4.63 or 9.26 kilometers. This mapping method offers a comprehensive perspective of SST variations across diverse geographical areas ^16^.

The World Ocean Circulation Experiment (WOCE) program was an ambitious international oceanographic research initiative conducted in the 1990s. With the participation of multiple countries and scientific organizations, the WOCE has focused on mapping and understanding global ocean circulation in detail. Fundamental technical data were collected through an extensive network of buoys, floats, and research vessel expeditions, including measurements of temperature, salinity, ocean currents, and sea level across the oceans. These data provide crucial information for understanding weather patterns, heat distribution on Earth, and ocean current variability, contributing significantly to the improvement of global climate models ^17, 18^.

**Figure 4.**
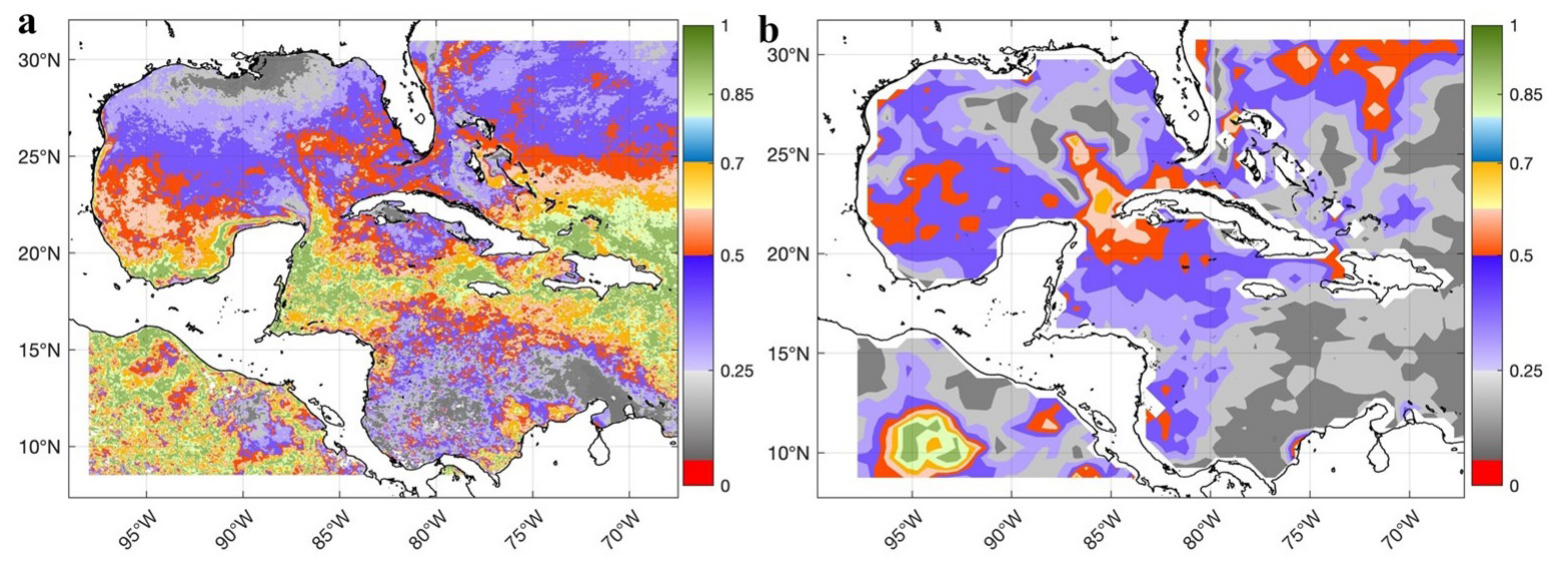
Validation of the SST of the GLOBAL_MULTIYEAR_PHY_001_030 product in the Caribbean Sea and the Gulf of Mexico through the spatial distribution of the p-values of the Mann-Whitney test for a significance level of 0.05: **a**) Comparison of the SST of the GLOBAL_MULTIYEAR_PHY_001_030 product with the SST of MODIS Aqua level 3 (Probability) and **b**) Comparison of the SST of the GLOBAL_MULTIYEAR_PHY_001_030 product with the SST of the WOCE dataset (Probability) ^20^.

The *GLOBAL_MULTIYEAR_PHY_001_030 product* is a reanalysis dataset; therefore, it must be validated against observed datasets, and the quality information document for this product must be reviewed ^19^. For surface validation, DSCompare v2.1 software was used ^20^, and the SST was validated using the Mann-Whitney statistical test comparing the global-reanalysis-phy-001-030 product with the observed MODIS Aqua level 3 and WOCE datasets. In both comparisons, no significant differences were found throughout the Caribbean Sea and Gulf of Mexico ( Figure 4); the significance level used was 0.05.

Drévillon ^19^ performed several validations of the GLOBAL_MULTIYEAR_PHY_001_030 reanalysis product, it has regional biases of less than 0.4 °C in temperature with respect to the World Ocean Atlas 2013 climatology (comparisons were made near surface, 100m, 300m, 500m, 800m, and 2000m depth) and to in situ observed data; the computed bias was less than 0.1 °C global mean with respect to in situ temperatures. The largest biases occurred in the 50–100m layer north of the Atlantic Ocean. The thermal structure improved significantly after 2002 with the deployment of Argo buoys, mainly at depths shallower than 300m, with an RMSE versus all in situ observations of less than 1°C and a bias close to 0°C and. The RMSE also decreases with time as a function of the density of the network of observations ^19^. The differences between the GLOBAL_MULTIYEAR_PHY_001_030 product and observations show that the reanalysis is very stable over the period 2000–2016. There is a small bias of 0 - 0.1°C located between 100 and 200m. This reanalysis product outperforms climatology in terms of bias and RMS, with lower RMS differences in the 0–500m layer ^19^. In addition, comparisons of this product with observed data from the CORA5 dataset ^21, 22^ and other sources in the years 1993, 1998, 2003, 2008, 2013, and 2016 showed that the mean differences tended to 0 °C in the Caribbean region.

The thermal efficiency of the ocean is closely linked to the heat exchange between the atmosphere and ocean. Large SST anomalies indicate significant deviations from their mean values, which can lead to a more substantial heat exchange; thus, the accuracy of the thermal efficiency computation can be affected. Large SST anomalies can also trigger feedback mechanisms in the climate system, further complicating the computation of ocean thermal efficiency. For example, a positive SST anomaly can lead to increased evaporation, which in turn affects heat fluxes and vertical temperature variation in the oceans. These anomalies can also affect ocean circulation patterns. These changes affect heat transport within the ocean, which in turn influences the computation of thermal efficiency.

**Figure 5.**
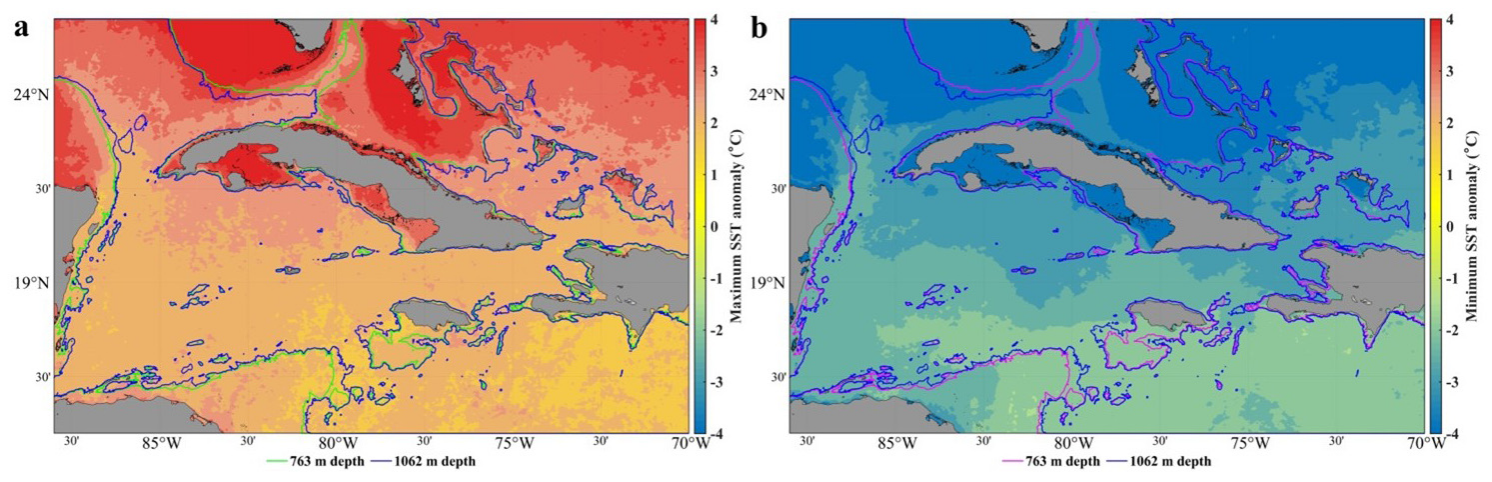
SST anomaly in the seawaters around Cuba ^23^, extracted from the DACS-PHY dataset ^24^, and computed with the CalcPlotAnomaly algorithm ^25^. **a**) Minimum SST anomalies. **b**) Maximum SST anomalies. 763 m and 1062 m isobaths taken from the GEBCO Bathymetric Compilation Group ^13^ **under** GEBCO 2018 Licence.

Figure 5 shows the minimum and maximum SST anomalies. The variability of the SST is greater in shallow waters, mainly in the gulfs (Guacanayabo, Batabano, and Ana Maria; see Figure 3), and in the archipelagos of southern and northern Cuba. In these areas, the depth is less than 763m, so this dataset does not provide thermal efficiency data. In the Bahama Bank and in some areas of the Gulf of Mexico the same thing happens, the SST variability is large, but depths shallower than 763m predominate. The SST anomaly varies less (the temperature is more stable) in the Caribbean Sea, in northern areas of Cuba (eastern and western part), in the Yucatan Channel, and in the Windward Passage ( Figure 3), where the depth is greater than 763m; therefore, the value of the thermal efficiency is quite accurate. The north of the central part of Cuba, north of the archipelagos (St. Nicolas Channel and Old Bahama Channel; see Figure 3), the temperature is more stable, but the depth does not reach 763m.

### Dataset

**Figure 6.**
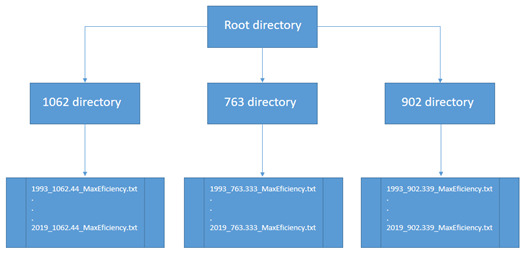
File distribution by directory.

The database for the calculation of thermal efficiency in the seas around Cuba is the result of an investigation conducted by ^1^. As shown in Figure 6, the dataset includes three folders that contain annual information on thermal efficiency from 1993 to 2019:

(i)  27 files calculated for the 1062m depth level.

(ii)  27 files, calculated for a depth level of 763m, and

(iii)  27 files calculated for the 902m depth level.

Each of the 81 files is stored in comma-separated txt format and 162.68 MB in size.

The same nodes of the regular data mesh of the temperature files **global-reanalysis-phy-001-030-daily** ^1^ for the study area were downloaded from the Copernicus site, and the number of nodes was 145 × 67. A description of the fields of each file separated by commas that do not have a header is presented in Table 1.

**Table 1.** Description of the fields of the output files of the calculation of thermal efficiency. *When it is not possible to calculate the thermal efficiency, either because the point is on land or because the depth level does not exist, the value is -32767.

Description of field	Data type
Date	Date
Level	float
Longitude	float
Latitude	float
Thermal efficiency	float *

## Ethics and consent

Ethics and consent were not required for this study

## Data Availability

The data is available on the Science Data Bank site. The site is free to download and use data sets respecting the corresponding data license. Science Data Bank: **Thermal Efficiency Dataset Around Cuban Seas (TEDACS)** https://doi.org/10.57760/sciencedb.10037 This project contains the following data: -  *_1062.44_MaxEficiency.txt -  *_763.333_MaxEficiency.txt -  *_902.339_MaxEficiency.txt *Years: 1993, 1994, 1995 … 2019 Data are made available under the terms of the Creative Commons Attribution 4.0 International License (CC-BY 4.0).
