# Thermal efficiency dataset around Cuban seas (TEDACS)

**DOI:** 10.12688/openreseurope.16815.2

**Published:** 2024-12-18

**Authors:** Alejandro Rodriguez, Melissa Abreu, Dailin Reyes, Melany Abreu, Humberto L. Varona, Carlos Noriega, Amilcar Calzada, Moacyr Araujo

**Affiliations:** 1 Marine Meteorology Center (CMM), National Institute of Meteorology (INSMET)., Havana, Havana, 11700, Cuba; 2 Laboratory of Physical, Coastal and Estuarine Oceanography (LOFEC)., Federal University of Pernambuco, Pernambuco, Recife-PE, Brazil; 3 Department of Oceanography (DOCEAN)., Federal University of Pernambuco. Recife-PE. Brazil., Pernambuco, Recife-PE, Brazil; 4 Center for Risk Analysis, Reliability Engineering and Environmental Modeling (CEERMA)., Federal University of Pernambuco. Recife-PE. Brazil., Pernambuco, Recife-PE, Brazil; 5 Brazilian Research Network on Global Climate Change (Rede CLIMA)., São José dos Campos, São Paulo, Brazil

**Keywords:** Thermal efficiency, OTEC technologies, Sea thermal energy, Sea Surface Temperature

## Abstract

Currently, the generation of electrical energy in Cuba is supported by oil and natural gas. These sources, as it is known, are directly linked to large emissions of pollutants that are released into the environment. Therefore, it is necessary to search for new energy options that are directed towards sustainable development, allowing the preservation of natural ecosystems. Owing to the location and geographical characteristics of Cuba, it is necessary to assess the energy possibilities of the seas that surround it and to search for the most feasible areas to obtain energy from the sea temperature. This renewable energy source, in addition to being used to generate electricity, can also be used in derived technologies, such as desalination, refrigeration, and aquaculture. Hence, a dataset is presented with the calculation of the Carnot thermal efficiency for the exploitation of thermal energy from the sea, which is based on the thermal gradient between the sea potential temperatures between the shore and the level of depth being analyzed. Outputs of 27 years of daily data from the Copernicus Marine Environmental Monitoring Service (CMEMS) GLOBAL_MULTIYEAR_PHY_001_030 product with a spatial resolution of 1/12° were used. The calculation was made using a Python script of the daily thermal efficiency at depths of 763, 902, and 1062 m, these depths belong to the depth levels of the model output data used according to the depth ranges that traditionally are studied for the exploitation of sea thermal energy. In this way, 27 files of each level were generated for a total of 81 files in text format separated by commas. Each file is presented with the date, level, coordinates, and thermal efficiency. The dataset is available from the Science Data Bank repository ( https://doi.org/10.57760/sciencedb.10037).

## Introduction

Ocean thermal energy conversion (OTEC) is a renewable energy technology that harnesses the solar energy absorbed by oceans to generate electricity. Heat from the sun warms surface water more than deep ocean water, creating a naturally available temperature gradient in the ocean. OTEC uses warm sea surface water with a temperature of approximately 25°C to vaporize a working fluid, which has a low boiling point, such as ammonia and propane. The vapor expands and turns a turbine coupled with a generator to produce electricity ^1^. The steam was then cooled with seawater that has been pumped up from the deepest layer of the ocean, where the temperature was approximately 5°C.

Thermal efficiency is the amount of heat/power used in a Rankine cycle for converting oceanic thermal energy into electrical energy ^2^. Among the different approaches used for the analysis of thermal energy efficiency, an approach based on a theoretical limit is used to maximize the efficiency of an OTEC system by converting the heat stored in the warm surface waters of the oceans into mechanical work ^1^.

According to previous studies, the temperature of the sea surface around Cuba oscillates throughout the year between 26° and 30°, on average. These characteristics indicate the convenience of studying the thermal energy of the seas around Cuba ^3^.

OTEC is a clean and friendly renewable energy with zero emissions, being capable of generating electricity 24 hours a day all year round, providing a reliable source of electricity. Also, multiple advantages are known, allowing to provide food for aquaculture farms, desalination (purification) of seawater, air conditioning of buildings, etc. This technology has been installed for research and development purposes in several regions of the world ^4^. Examples of the aforementioned are Saga and Kumejima (Japan), Reunion Island (France), Gosung (South Korea) and Hawaii (USA). Likewise, studies have been carried out on the energy potential of sea thermal energy in Indonesia, Malaysia, the Philippines ^4– 6^, the Gulf of Mexico the Caribbean ^7, 8^ and the Mexican Pacific ^9^.

The present work exposes the main features of a dataset that contains the computation of Carnot thermal efficiency in the seas surrounding Cuba between latitudes 18.5° and 24.0° and longitudes -73.5° and -85.5°. The objective is to evaluate the study area according to the requirements for obtaining thermal energy from the sea, which must reach values greater than or equal to 0.7 of thermal efficiency ^10^. For the computation, 27 years of global ocean reanalysis data from the GLOBAL_MULTIYEAR_PHY_001_030 product of the Copernicus Marine Environmental Monitoring Service (CMEMS) with a spatial resolution of 1/12° were used ^11^.

With this dataset presented, it is also possible to develop other investigations in the study area to advance the knowledge of the thermal energy potential of the sea to investigate the selection of sites where OTEC plants can be built for the generation of electrical energy, desalination seawater, and other applications.

In the following sections, the methods are presented, which introduce the study area, equation for calculating thermal efficiency, data validation, vertical temperature assessment; dataset with its structure, generated files, and availability of data.

## Methods

This study was performed using the output cmems_mod_glo_phy_my_0.083_P1D-m, available for free download from the Copernicus Marine Environment Monitoring Service (CMEMS) GLOBAL_MULTIYEAR_PHY_001_030 ( https://data.marine.copernicus.eu/product/GLOBAL_MULTIYEAR_PHY_001_030/services), that simulates the global ocean at 1/12° (approximately 8 km) resolution ^11^, using the space-time evolution of the 3D thermodynamic variables temperature and salinity (T, S) at 4 levels: -0.49, -763.33, -902.34 and -1062.44 m. Were downloaded 27 years of these files with the "motuclient-python" tool ^12^, which were then manipulated and analyzed with the CDO **adisit** function ^13^, to calculate the temperature in situ from potential temperature and salinity data.

Using the nodes from the data files to calculate Carnot thermal efficiency, the depth levels from these files were also used. The cold sea temperature at a depth of 1000 meters is used as a standard for calculating the ocean temperature gradient. Therefore, according to the depth levels of the reanalysis files used, the level 1062.44 meters is the closest to 1000 meters. On the other hand, due to the high temperatures of the sea surface around Cuba, it was decided to also do the calculation for the 900 and 800 meters depth; Coincident as the closest depth levels are the levels of 902,339 and 763,333 respectively.

The calculations of the maximum efficiency and the generation of the efficiency maps for each level were performed using Python version 3.9 (RRID:SCR_008394) of Python language. Therefore, the presented dataset contains the computation of thermal efficiency in the seas surrounding Cuba between latitudes 18.5° and 24.0° and longitudes -73.5° and -85.5° ^10^.

## Brief characterization of the study area

The study area is influenced by the marine current systems that surround it: The Caribbean current, which extends from the arc of the Lesser Antilles to the vicinity of the Yucatan Peninsula; the Yucatan Current, which connects the Caribbean Sea and the Gulf of Mexico; the Loop Current, a flow that joins the Yucatan current and the La Florida current in the eastern part of the Gulf of Mexico; and the Florida Current, from the Straits of Florida to Cape Hatteras, and is considered the beginning of the Gulf Stream. Trend studies have observed that marine flow in the Caribbean Sea has had a slight decrease in magnitude, while in the Gulf of Mexico and the Near Atlantic, there has been a subtle increase ^14^.

**Figure 1.**
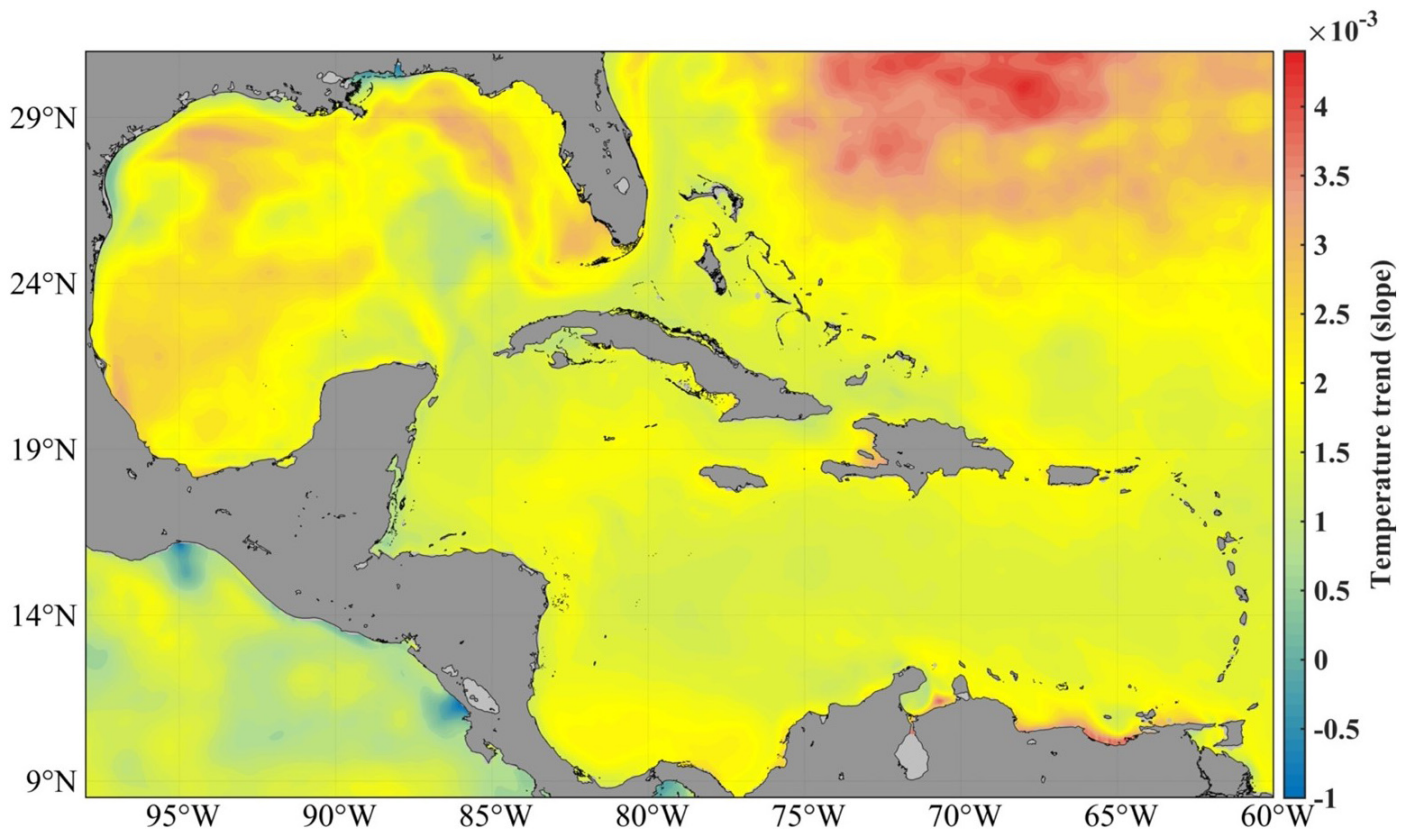
SST trend from GLOBAL_MULTIYEAR_PHY_001_030 product, 1993–2018 ^11^.

In this area, the average temperature of the sea surface oscillates between 25 and 30º, with a minimum in February and a maximum in September. The maxima are found over deep waters in the area of the Casilda-Cazones Gulf and south of Isla de la Juventud, towards the central and northern Caribbean area, as well as another maximum north of the western coasts. Extreme minimum values were recorded in the Gulf of Mexico area, with 24° and maximums values of up to 31°. Calculation of the sea surface temperature trend with the reanalysis data used in this research revealed an increase between 0.5° and 2.5° ( Figure 1).

**Figure 2.**
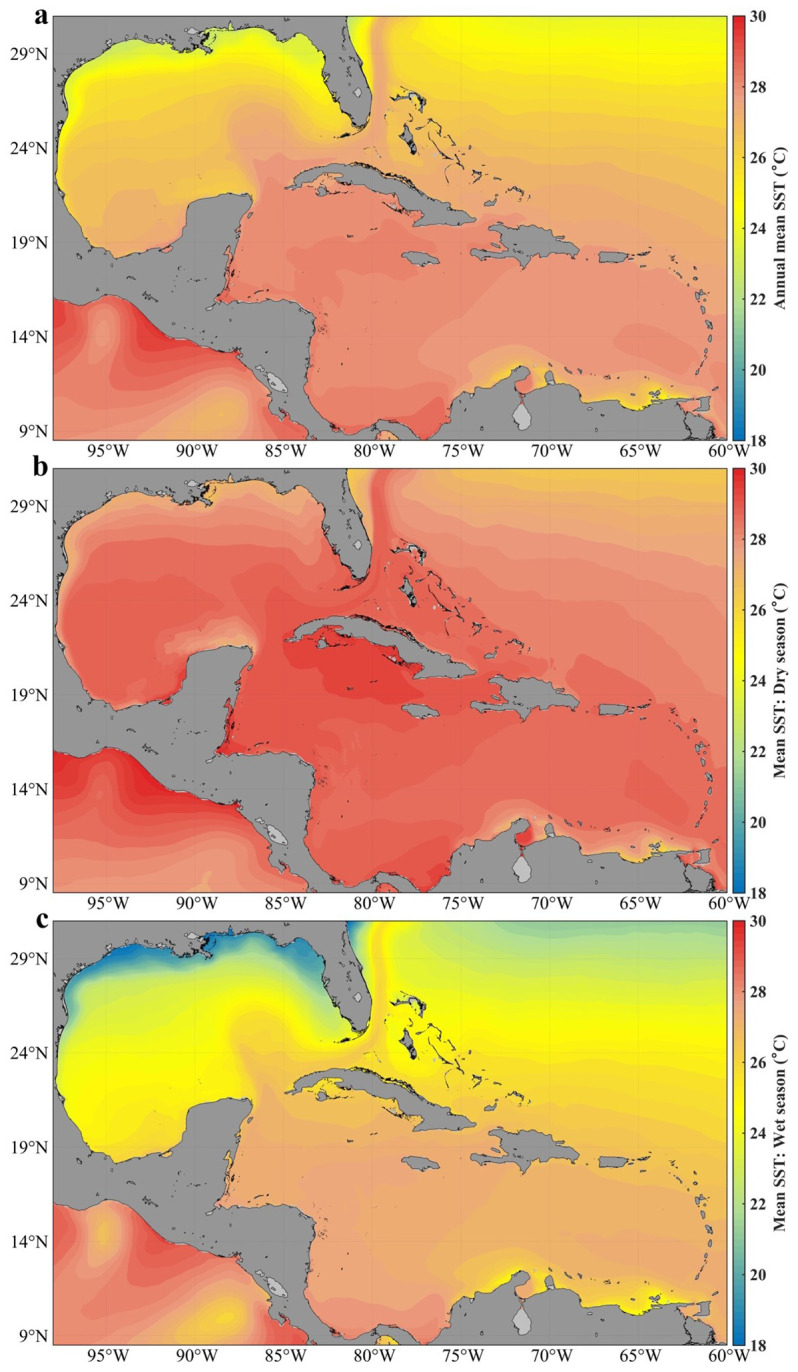
Analysis of the mean sea surface temperature in Cuban waters with the GLOBAL_MULTIYEAR_PHY_001_030 product in the Caribbean Sea and the Gulf of Mexico ^11^: **a**) distribution of the annual mean, **b**) distribution in the dry season, **c**) distribution in the wet season.

The area is also affected by teleconnection events such as the NAO and ENSO, showing a zonal pattern in the correlation between the sea surface temperature and the NAO index six months before the development of the negative and positive phases of the event ^15^. In addition, extreme phenomena such as hurricanes and cold fronts affect the Cuban archipelago every year, highlighting the relationship between the intensity of these phenomena and the different phases of the NAO and ENSO ^16^. Figure 2a shows the annual spatial distribution of sea surface temperature (SST) in Cuban waters, while Figure 2b and c show the seasonal spatial distribution.

## Bathymetric characterization

**Figure 3.**
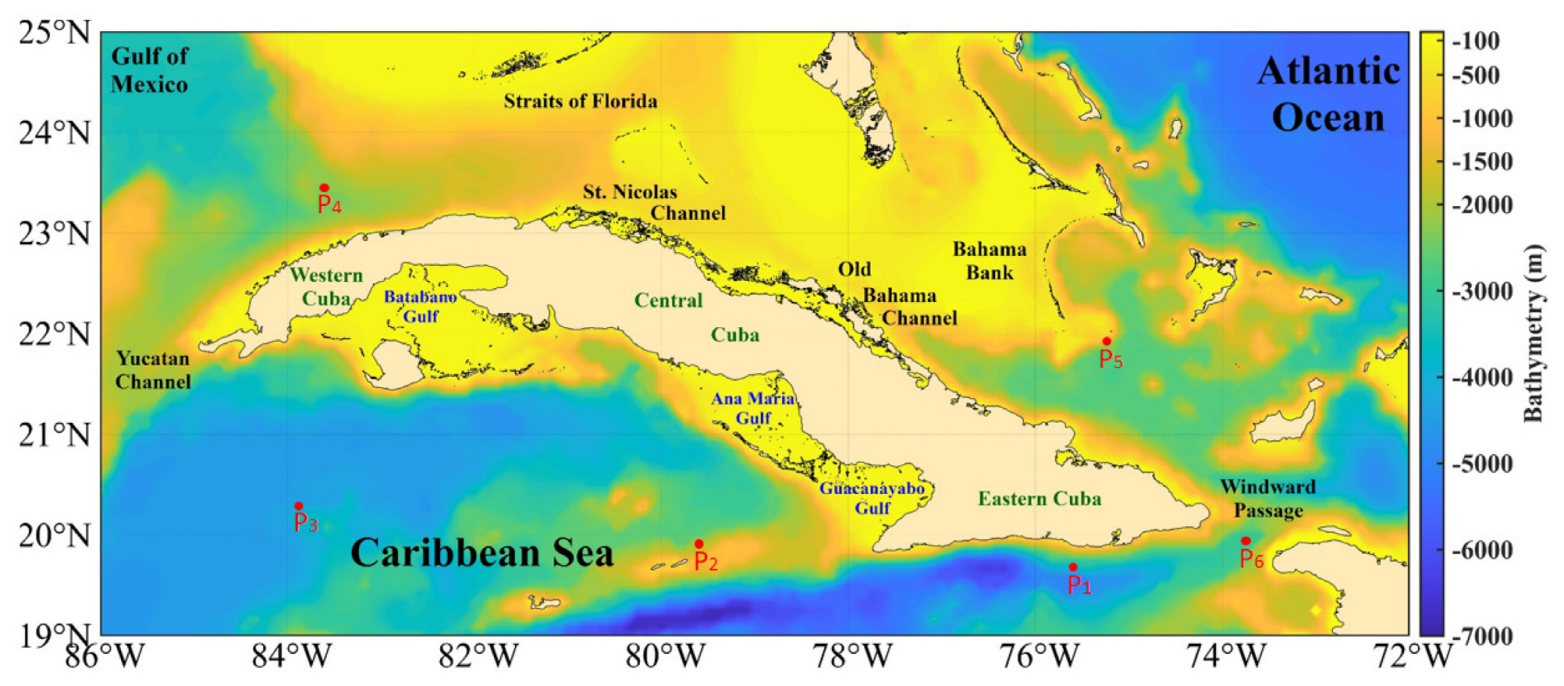
Bathymetry of the seawaters around Cuba taken from the GEBCO Bathymetric Compilation Group ^19^ under GEBCO 2018 Licence. P1, P2, P3, P4, P5, and P6 are the points selected for vertical temperature assessment.

Cuba is geographically located in the American Mediterranean, between longitudes 74°7'52" W and 84°54'57" W, and latitudes 19°49'36" N and 23°17'9" N ( Figure 3). It is bordered to the north by the Gulf of Mexico, Florida Strait, St. Nicholas Channel, and old Bahama Channel; to the south by the western Caribbean Sea and Strait of Columbus; to the west by the Yucatan Channel; and to the east by the Windward Passage ^17^. The westernmost and northernmost island platforms contain the Guanahacabibes Gulf, with depths of 5–25m. Less than 1 mile from the outer edge, the shelf's insular slope drops steeply from 10 to 100m. Reefs appear parallel to the coast, cut by numerous ravines, and pass between the 5 and 10m isobaths, which form a chain that obstructs access to the coast. Continuing along the western north of the Cuban archipelago, the insular slope presents an abrupt, steep, and sinuous drop, very close to the coast, which causes the 200m isobath to be frequently found less than 1 mile from the slope ^18^.

The island platform, in the northern central zone (from Punta Hicacos to Punta Maternillos), is wide, and its edge generally registers depths between 10–25m; outside the edge of the platform, the depths increase sharply, so the island slope varies from to 20–200m depth in less than 1m of distance. Above this island platform, the most continuous and extensive coral barrier in Cuba extends, with depths of less than 10m and in many places less than 5m, with cays and heads that emerge. In the northeastern area (from Nuevitas Bay to Punta Maisí), the island platform, less than 10m in depth, has an edge of 3 miles from the coast. Outside the island shore, there are no shallows, heads, or reefs that are dangerous to navigation, and the depths increase abruptly; the 200m isobath crosses within one mile of the border throughout the entire region ^18^.

Continuing along the southeastern area, the entire coast is bordered by a low strait of less than 20m depth, whose edge corresponds to the vertical slope that the Eastern Trench presents in this part of the coast. The 200m isobath crosses very close to the coast, over an underwater relief that reaches great depths to the south, so the island platform of the Island of Cuba becomes very narrow, and the slope falls abruptly towards abyssal depths where it is located. The Eastern trench reaches 7,239m depth. Further to the west south, in the approaches to the Guacanayabo Gulf from the sea, the depths are great and the 200m isobath crosses 1.6 miles southwest of Cay Cruz. The isobath is the edge of the island platform, made up of coral reefs submerged at a depth of 20–40m. Among these reefs, depths are generally less than 20m, with a gentle slope ^18^.

Continuing westwards to the south, the Ana María Gulf is separated from the Guacanayabo Gulf by the cays Mate and Laberinto de las Doce Leguas ( Figure 3); and to the southwest it is limited by the barrier reef of the Jardines de la Reina Archipelago, which extends 118 miles to the northwest with depths mostly less than 5m. The greatest distance between the edge of the platform and the coral reef barrier does not exceed 2 miles. Beyond this, the depths are abyssal, located at distances between 16 and 20 miles, with depths of 22–58m. From the Punta María Aguilar to the Cazones Gulf, the shallow area at less than 10m depth that borders the coast is very narrow, and there are places where the cliffed coast falls directly to greater depths. Depths of 100m were recorded 2 miles from the coast and 2,000m in the central part of the Gulf of Cazones ^18^.

Next, from south to west, the underwater relief of the Batabanó Gulf has some irregularities due to the existence of cays and shoals with depths between 2–5m ( Figure 3). In the central part, the underwater relief of the gulf is gentle, with maximum depths between 6–7m, and a shallow slope. In the westernmost area of the southern portion, from Guano del Este Cay to San Antonio Cape, the slope of the island platform of Cuba falls abruptly towards great depths, finding in many places an isobath of 1,000m very close to the edge of the platform. Finally, the southern and southeastern coasts of Isla de la Juventud, with steep cliffs, drop abruptly to abyssal depths of 3,000m ^18^.

## Computation of thermal efficiency

The real thermal efficiency ( *η*_real_) is obtained by multiplying the Carnot efficiency by the efficiencies of the individual components and subtracting the losses, therefore, an equation of a fairly complete general form can be written to determine the real thermal efficiency as follows: this equation can be written as a general way to compute the real thermal efficiency ( Equation 1).

$$\eta_{\text{real}}=\eta_{\text{Carnot}}\times \eta_{Exchanger}\times \eta_{\text{Turbine}}\times \eta_{\text{Generator}}-\frac{P_{\text{Pumps}}+P_{\text{Auxiliaries}}}{Q_{\text{Input}}}\;(1)$$

Where *η*_Carnot_ is the theoretical maximum efficiency of a Carnot cycle ( Equation 2) ^20^.

$$\;\eta_{\text{Carnot}}=1-\frac{T_{Cold}}{T_{\text{Warm}}}\;(2)$$

*T*_Warm_ is the absolute temperature of the hot source (surface water), *T _Cold_* is the absolute temperature of the cold source (deep water), *η _Exchanger_* measures the ability of a heat exchanger to transfer heat between two fluids at different temperatures. It is the ratio of the actual heat transferred to the maximum theoretically possible heat transfer ^21^, *η*_Turbine_ indicates how effectively the turbine converts the thermal or pressure energy of the working fluid into rotational mechanical energy ^22, 23^, *η*_Generator_ measures how effectively the generator converts mechanical rotational energy into electrical energy, *Q*_Input_ represents the heat absorbed by the system from the hot source, in this case, the surface ocean water, *P*_Pumps_ is the electrical power consumed by the pumps used to move water from and to different depths in the ocean, and *P*_Auxiliaries_ represents the power consumed by all auxiliary systems that support the main operation but do not directly contribute to energy production ^24, 25^.

The equation for η _real_ as the product of several individual efficiencies is derived by analyzing the contributions of each system component to the overall efficiency ^26, 27^. Mathematically, this is based on how the available energy is distributed throughout the different stages of the process. The theoretical and practical underpinnings provided for each component of Equation 1 were taken from El-Wakil ^28^, Incropera *et al.* ^21^, Karassik *et al.* ^24^, Moran and Shapiro ^20^, Chapman ^29^, Dixon and Hall ^22^, Yuan *et al.* ^23^, Çengel and Boles ^25^, Zhang *et al.* ^26^, Fontaine *et al.* ^30^, and Abbas *et al*. ^27^.

In the most feasible marine areas for the operation of OTEC plants, the average surface temperature of each year is approximately 26.7 to 29.4°, with cold water available at 4.4° or less at a depth of 900–1000m ^20^. Therefore, even without the inevitable reduction caused by friction and heat loss, the maximum efficiency achieved by heat conversion in an OTEC plant can be achieved with a very small rate of energy production. The objective of this research is to study the thermal efficiency of a Carnot cycle. To apply the calculation of thermal efficiency, the approach proposed in the research carried out in was considered, which is based on a theoretical limit, up to a maximum efficiency of an OTEC system through the conversion of heat into mechanical work stored in the warm surface waters of the tropical oceans ( Equation 2).

The computation of the Carnot thermal efficiency around Cuba ^31^ was made without taking into account the characteristics of the coastal relief at the selected points. The calculation process with the data used is described with the following steps:

a. Using the CDO adisit function, the potential temperature from the reanalysis file was converted to in situ temperature.

b. The study area was divided into 2 zones: northern zone and southern zone.

c. The points on land that had the geographic longitude values of the nodes in the reanalysis file for each zone were located, assigning each one the surface temperature value of the closest node and with the same geographic longitude value to the selected coastal point.

d. The calculation of thermal efficiency with the Equation (1) was applied to each node with temperature values at each depth selected for the study, moving in a north (south) direction for the north zone (southern zone).

## Vertical temperature assessment

The evaluation of the vertical temperature will be performed by comparing the vertical temperature profiles at 6 points obtained from the GLOBAL_MULTIYEAR_PHY_001_030 product (re-analysis) ^24^, whose geographical location is shown in Figure 3 and Table 1. The reference vertical profiles were obtained from the MULTIOBS_GLO_PHY_TSUV_3D_MYNRT_015_012 product ^12, 32^ at the same coordinates. The monthly bias is calculated by Equation 3 ^33^ using the vertComp software ^34^.

$$\;Bias=\frac{\Sigma_{i=1}^{n}(D_{i}-R_{i})}{n}\;(3)$$

Where *D _i_* are the values of the reference dataset, *R _i_* are the values of the re-analysis dataset, and *n* is the total of months for which the comparison was made. The MULTIOBS_GLO_PHY_TSUV_3D_MYNRT_015_012 product, developed by the CLS Production Unit (MULTIOBS-CLS-TOULOUSE-FR), offers global Level-4 (L4) analyses of oceanic 3D temperature, salinity, geopotential height, and geostrophic currents from the surface to a depth of 5500 meters, along with 2D Mixed Layer Depth (MLD) on a 1/4° regular grid. The temperature biases are minimal, particularly during the 2004-2018 Argo period. The root mean square differences range from 0.8°C to 1.4°C at 100 meters depth and from 0.18°C to 0.5°C at 1000 meters depth. The expansion of the Argo network does not cause inhomogeneity in ARMOR3D, and the trends and climatic modes remain consistent despite the changes. The thermosteric trend in dynamic height for the [0-1500 m] layer between 1993 and 2018 is approximately 1 mm/year. This trend has not yet been recalculated for the entire period (as of the publication date) but will be updated in the next complete reprocessing revision before the end of 2024.

**Table 1.** Geographical location of the points used for temperature assessment of GLOBAL_MULTIYEAR_PHY_001_030 product.

Name	Longitude	Latitude
P1	75.67 °W	19.75 °N
P2	79.67 °W	19.92 °N
P3	83.92 °W	20.33 °N
P4	83.83 °W	23.50 °N
P5	75.33 °W	21.67 °N
P6	73.75 °W	20.08 °N

## Analysis of uncertainty in the spatial and temporal distribution of temperature

The Median Absolute Deviation (MAD) is a measure of statistical dispersion that indicates the average distance between each data point and the median of the dataset. It is a robust measure of spread ^35^, meaning it is less sensitive to outliers compared to the standard deviation. The MAD provides a measure of dispersion or spread of ocean temperatures around the median value. A lower MAD indicates that the temperatures are clustered closer to the median, suggesting a more uniform distribution. Conversely, a higher MAD suggests that the temperatures ( *T*) are more dispersed, indicating greater variability. Analyzing MAD across different ocean regions can reveal patterns in temperature variability. For instance, regions with lower MADs might experience more stable temperature conditions, while regions with higher MADs might exhibit greater fluctuations. The MAD is computed using the mathematical Equation 4.

MAD(T)=Median|T−Median(T)|(4)

The MAD is useful for analysing data uncertainty because it is a robust measure of variability ^36– 38^, less affected by outliers compared to standard deviation. By relying on the median rather than the mean, MAD provides a more accurate representation of standard deviation in data sets with outliers. This robustness makes it ideal for assessing uncertainty in situations where data may not follow a normal distribution, ensuring a more reliable estimate of variability. MAD can also be used to identify outliers ^39^, as data points with large absolute differences from the median are potential outliers ^40^; to compare the dispersion of two or more datasets; and to analyze ordinal data, which is data that can be ranked but doesn't have meaningful numerical differences.

The temperature of the GLOBAL_MULTIYEAR_PHY_001_030 product are stored in 4D variable as T(lon, lat, depth, time), to compute the MAD we have set the depth at the surface, 763, 902, and 1062 m. Remaining in 3D as T(lon, lat, time) in 4 vertical levels, this simplifies the MAD computation. Thus, MAD at the surface can be calculated according to Equation (5).

$$MAD_{\lambda ,\varphi }^{z}\;=Median|T(\lambda ,\varphi ,z,t)-Median(T(\lambda ,\varphi ,z,t))|\;(5)$$

Where *λ* and *φ* are the longitudes and latitudes respectively and *t* is in time (monthly mean). *T* is the temperature and *SST* is the sea surface temperature, both in ° C. In Equation 4, Equation 5, and Equation 6, the depth is denoted by z and only values of *Z* = 0.5,763,902,1062 *m* are taken into account. It is also analysed how MAD varies with respect to longitude over time ( $MAD_{\lambda ,t}^{z}$ ; Equation (6) and with respect to latitude over time ( $MAD_{\varphi ,t}^{z}$ ; Equation (7). All these parameters will be computed through the gStat software ^39^.

$$\;MAD_{\lambda ,t}^{z}\;=Median|T(\varphi_{j},z,t_{k})-\text{Median}(T(\varphi_{j},z,t_{k}))|\;(6)$$

$$MAD_{\varphi ,t}^{z}\;=Median|T(\lambda_{i},z,t_{k})-Median(T(\lambda_{i},z,t_{k}))|\;(7)$$

## Data validation

The MODIS Aqua Level 3 dataset encompasses sea surface temperature (SST) information derived from the NASA MODIS sensor situated on the Aqua satellite. SST data were extracted from the thermal infrared spectrum with wavelengths of 11 and 12 μm. After processing, the data is projected onto a cylindrical equidistant map with spatial bins measuring either 4.63 or 9.26 kilometers. This mapping method offers a comprehensive perspective of SST variations across diverse geographical areas ^41^.

The World Ocean Circulation Experiment (WOCE) program was an ambitious international oceanographic research initiative conducted in the 1990s. With the participation of multiple countries and scientific organizations, the WOCE has focused on mapping and understanding global ocean circulation in detail. Fundamental technical data were collected through an extensive network of buoys, floats, and research vessel expeditions, including measurements of temperature, salinity, ocean currents, and sea level across the oceans. These data provide crucial information for understanding weather patterns, heat distribution on Earth, and ocean current variability, contributing significantly to the improvement of global climate models ^42, 43^.

**Figure 4.**
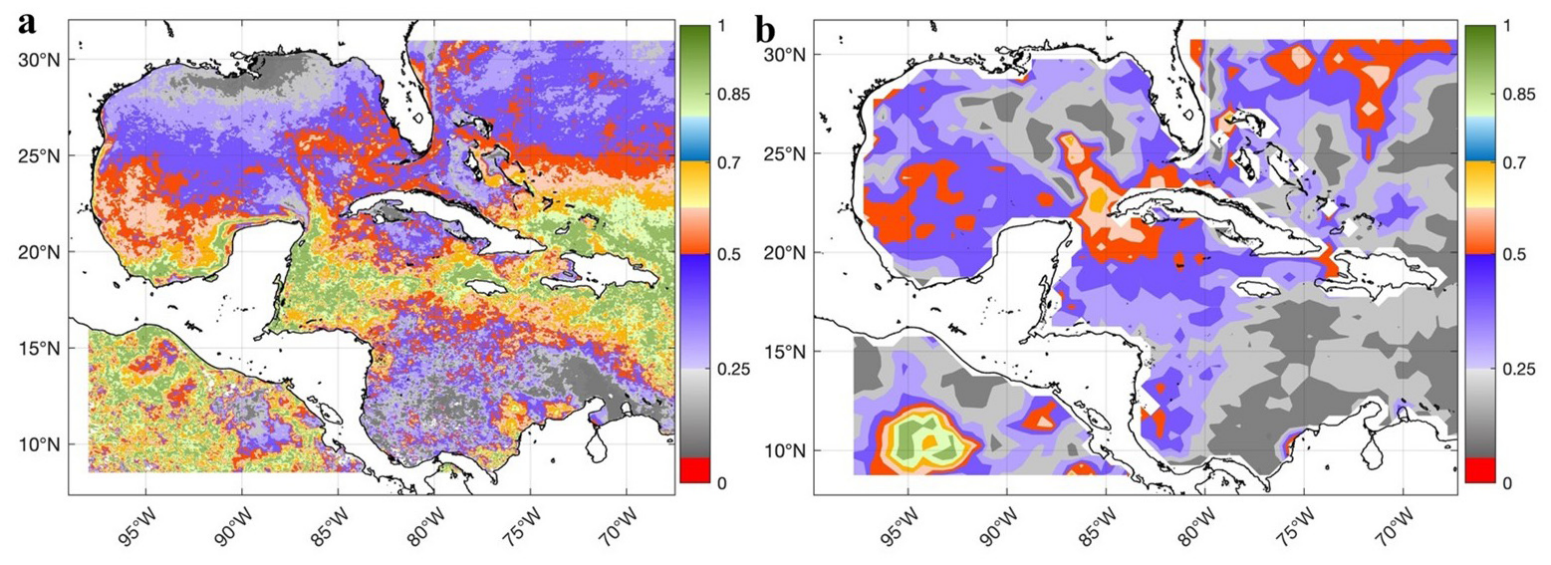
Validation of the SST of the GLOBAL_MULTIYEAR_PHY_001_030 product in the Caribbean Sea and the Gulf of Mexico through the spatial distribution of the p-values of the Mann-Whitney test for a significance level of 0.05: **a**) Comparison of the SST of the GLOBAL_MULTIYEAR_PHY_001_030 product with the SST of MODIS Aqua level 3 (Probability) and **b**) Comparison of the SST of the GLOBAL_MULTIYEAR_PHY_001_030 product with the SST of the WOCE dataset (Probability) ^32^.

The *GLOBAL_MULTIYEAR_PHY_001_030 product* is a reanalysis dataset; therefore, it must be validated against observed datasets, and the quality information document for this product must be reviewed ^44^. For surface validation, DSCompare v2.1 software was used ^35^, and the SST was validated using the Mann-Whitney statistical test comparing the global-reanalysis-phy-001-030 product with the observed MODIS Aqua level 3 and WOCE datasets. In both comparisons, no significant differences were found throughout the Caribbean Sea and Gulf of Mexico ( Figure 4); the significance level used was 0.05.

Drévillon ^44^ performed several validations of the GLOBAL_MULTIYEAR_PHY_001_030 reanalysis product, it has regional biases of less than 0.4 °C in temperature with respect to the World Ocean Atlas 2013 climatology (comparisons were made near surface, 100m, 300m, 500m, 800m, and 2000m depth) and to in situ observed data; the computed bias was less than 0.1 °C global mean with respect to in situ temperatures. The largest biases occurred in the 50–100m layer north of the Atlantic Ocean. The thermal structure improved significantly after 2002 with the deployment of Argo buoys, mainly at depths shallower than 300m, with an RMSE versus all in situ observations of less than 1°C and a bias close to 0°C and. The RMSE also decreases with time as a function of the density of the network of observations ^44^. The differences between the GLOBAL_MULTIYEAR_PHY_001_030 product and observations show that the reanalysis is very stable over the period 2000–2016. There is a small bias of 0 - 0.1°C located between 100 and 200m. This reanalysis product outperforms climatology in terms of bias and RMS, with lower RMS differences in the 0–500m layer ^44^. In addition, comparisons of this product with observed data from the CORA5 dataset ^45, 46^ and other sources in the years 1993, 1998, 2003, 2008, 2013, and 2016 showed that the mean differences tended to 0 °C in the Caribbean region.

The thermal efficiency of the ocean is closely linked to the heat exchange between the atmosphere and ocean. Large SST anomalies indicate significant deviations from their mean values, which can lead to a more substantial heat exchange; thus, the accuracy of the Carnot thermal efficiency computation can be affected. Large SST anomalies can also trigger feedback mechanisms in the climate system, further complicating the computation of ocean thermal efficiency. For example, a positive SST anomaly can lead to increased evaporation, which in turn affects heat fluxes and vertical temperature variation in the oceans. These anomalies can also affect ocean circulation patterns. These changes affect heat transport within the ocean, which in turn influences the computation of thermal efficiency.

**Figure 5.**
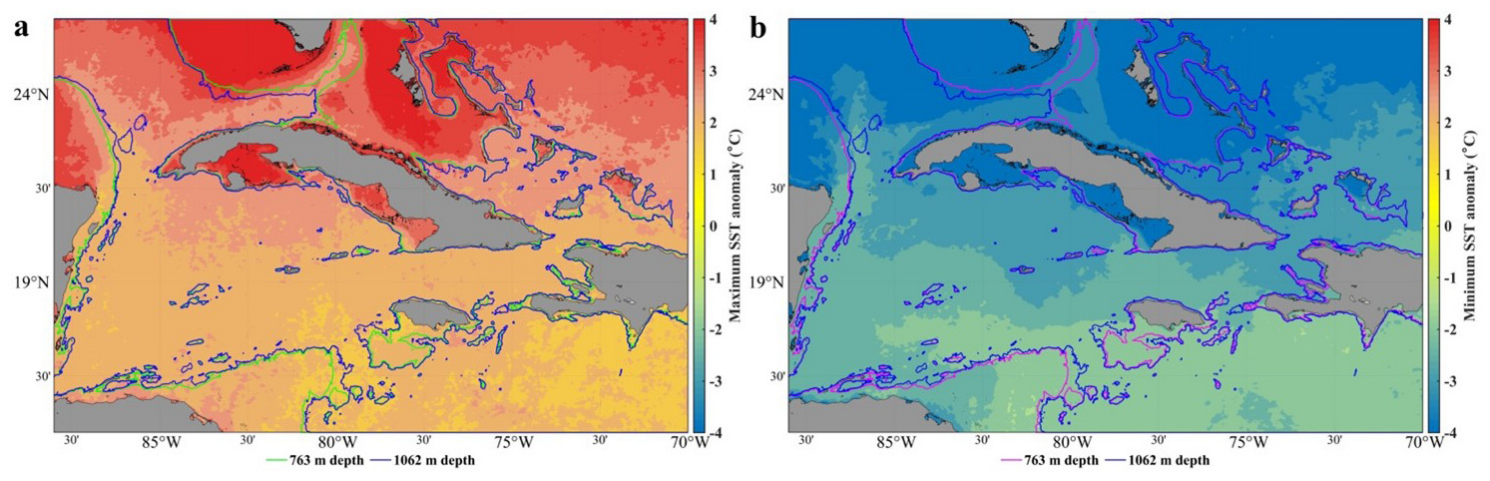
SST anomaly in the seawaters around Cuba ^35^, extracted from the DACS-PHY dataset ^36^, and computed with the CalcPlotAnomaly algorithm ^37^. **a**) Minimum SST anomalies. **b**) Maximum SST anomalies. 763 m and 1062 m isobaths taken from the GEBCO Bathymetric Compilation Group ^19^ **under** GEBCO 2018 Licence.

Figure 5 shows the minimum and maximum SST anomalies. The variability of the SST is greater in shallow waters, mainly in the gulfs (Guacanayabo, Batabano, and Ana Maria; see Figure 3), and in the archipelagos of southern and northern Cuba. In these areas, the depth is less than 763m, so this dataset does not provide thermal efficiency data. In the Bahama Bank and in some areas of the Gulf of Mexico the same thing happens, the SST variability is large, but depths shallower than 763m predominate. The SST anomaly varies less (the temperature is more stable) in the Caribbean Sea, in northern areas of Cuba (eastern and western part), in the Yucatan Channel, and in the Windward Passage ( Figure 3), where the depth is greater than 763m; therefore, the value of the thermal efficiency is quite accurate. The north of the central part of Cuba, north of the archipelagos (St. Nicolas Channel and Old Bahama Channel; see Figure 3), the temperature is more stable, but the depth does not reach 763m.

**Figure 6.**
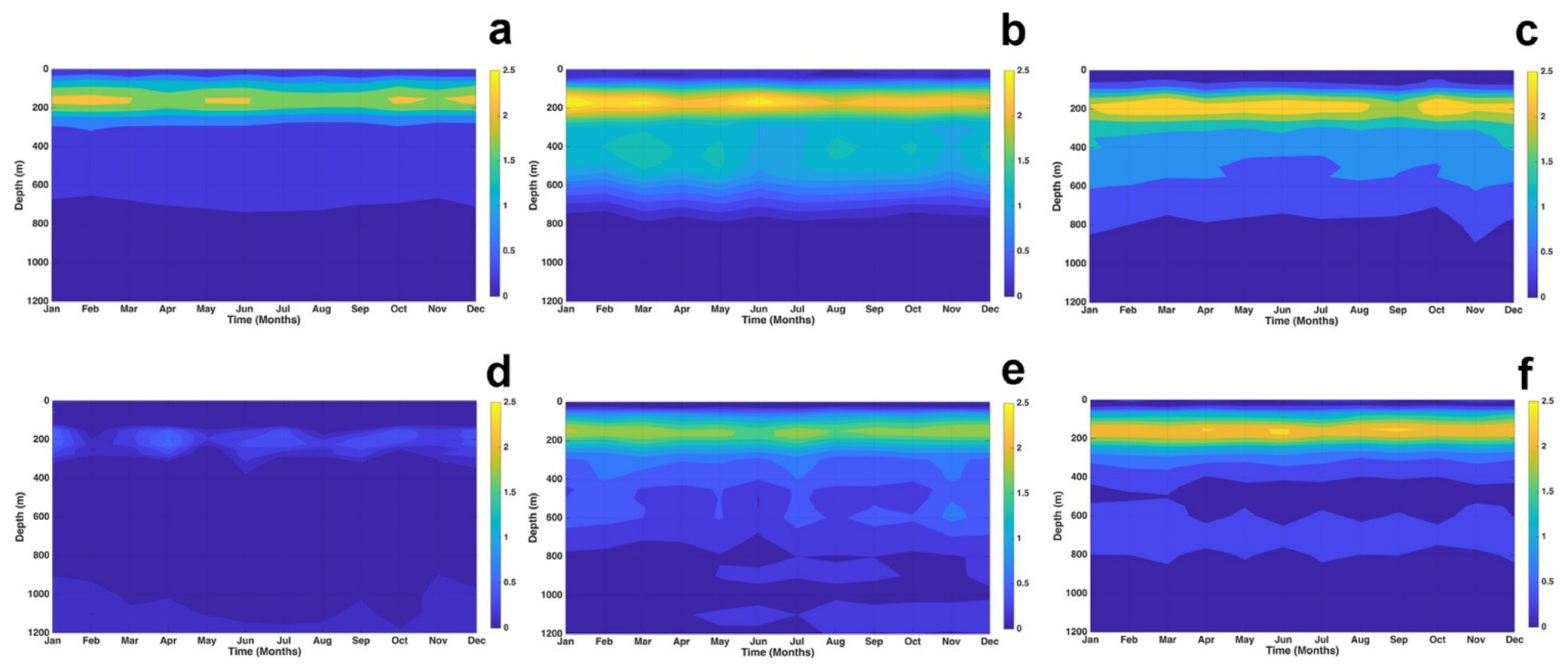
Evaluation of the vertical temperature of the product MULTIOBS_GLO_PHY_TSUV_3D_MYNRT_015_012 at points: **a**) P1, **b**) P2, **c**) P3, **d**) P4, **e**) P5, and **f**) P6.

Figure 6 shows the vertical comparison between the GLOBAL_MULTIYEAR_PHY_001_030 and MULTIOBS_GLO_PHY_TSUV_3D_MYNRT_015_012 products at the points located according to Table 1. In all profiles, good agreement is observed in the first 30 m depth, with bias less than 0.5 ° C. In P1, P2, P3 P5 and P6, in the layer from 30 to 250 m, the maximum biases are obtained, reaching to exceed 2 ° C in all months in P2, P3 and P6, while in P1 and P5, the bias varies between 1. 5 and 2 ° C. For depths greater than 250 m, a good agreement is obtained throughout the year in all profiles with a bias less than 0.75 ° C, existing in P4 an excellent agreement throughout the period in all depths. All this makes the GLOBAL_MULTIYEAR_PHY_001_030 product good for this study.

**Figure 7.**
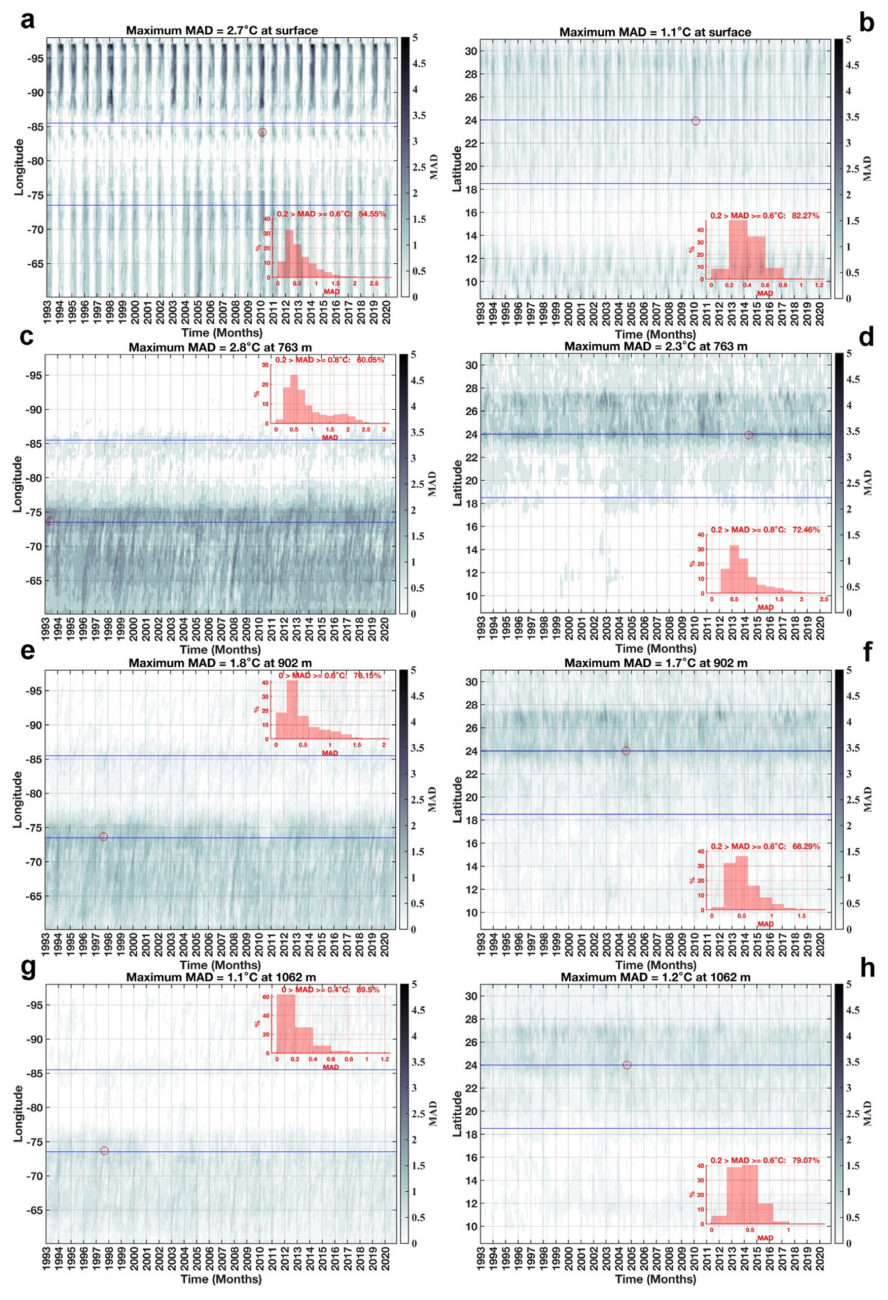
MAD distribution in longitude and latitude with respect to time: **a**) longitude vs time at surface, **b**) latitude vs time at surface, **c**) longitude vs time at 763 m, **d**) latitude vs time at 763 m, **e**) longitude vs time at 902 m, **f**) latitude vs time at 902 m, **g**) longitude vs time at 1062 m, and **h**) latitude vs time at 1062 m. The blue lines delimit the longitudes and latitudes of our study region. The red circle represents the maximum MAD within the study region. The histograms represent the percentage of MAD according to their values.

The MAD distribution over longitudes and latitudes with respect to time is shown in Figure 7. Analyzing the variation of SST in Figure 7ab, it was observed that SST values clustered around the median by 54.55% and 82.27% with respect to longitude and latitude over an interval of 0.2 > *MAD* ≥ 0.6° *C*. At 763 m depth, temperature values clustered around the median by 60.05% and 72.46% with respect to longitude and latitude in an 0.2 > *MAD* ≥ 0.8° *C* interval ( Figure 7cd). In these two layers, the latitudinal variations of temperature with respect to the median are smaller than the longitudinal variations, on the contrary, in layers 902 m ( Figure 7ef) and 1062 m ( Figure 7gh) the longitudinal variations of temperature around the median are smaller than the latitudinal variations. At 902 m the temperature values are clustered around the median by 76.15% and by 68.29% with respect to longitudes and latitudes in the intervals of 0 > *MAD* ≥ 0.6° *C* and 0.2 > *MAD* ≥ 0.6° *C* respectly, this behavior is similar to that occurring at 1062 m, where the clustering of temperature values around the median is 89.5% with respect to longitudes in interval 0 > *MAD* ≥ 0.4° *C* and 79.07% with respect to latitudes in interval 0.2 > *MAD* ≥ 0.6° *C*. All this indicates that in the two deeper layers, the temperature is more stable in the spatio-temporal variations.

**Figure 8.**
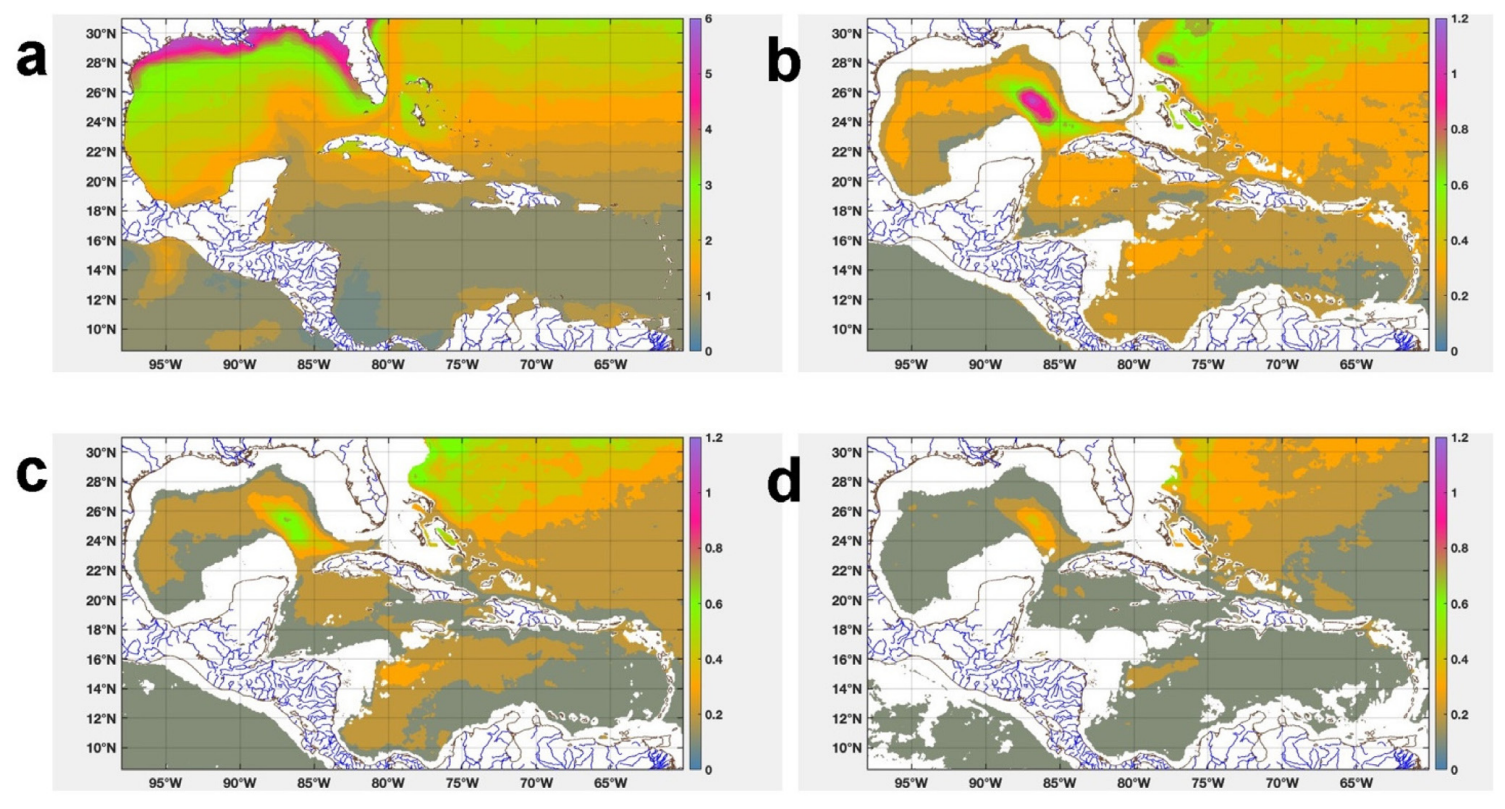
MAD spatial distribution of temperature: **a**) Surface, **b**) 763 m, **c**) 902 m, and **d**) 1062 m.

Figure 8 shows the spatial distribution of the MAD in the study region, in the four layers there is a clustering of the temperature values around the median. This clustering is smaller in the SST values, being in an interval of 0 > *MAD* ≥ 1.5° *C*, reaching values in the range of 1.5 > *MAD* ≥ 1.75° *C* in the Ana Maria and Guacanayabo Gulfs and MA = 2.4 ° C in the Batabano Gulf. In the rest of the layers the temperature values are clustered around the median in the interval of 0 > *MAD* ≥ 0.3° *C*. This figure also shows that the deeper the layer, the more stable the temperature is.

### Mapping thermal efficiency

**Figure 9.**
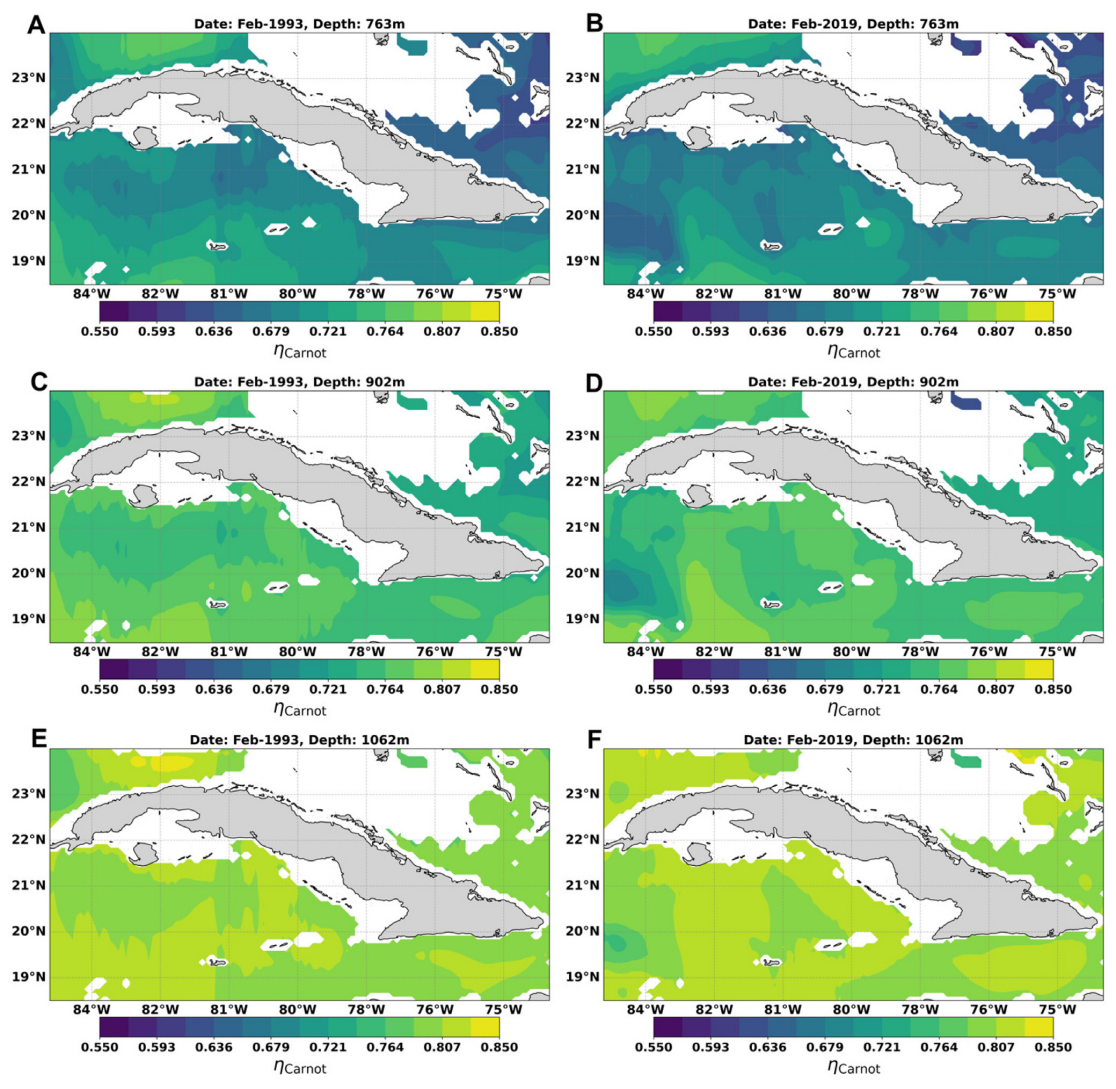
Spatial distribution of the theoretical maximum efficiency of a Carnot cycle in the waters adjacent to Cuba: **A**) Feb/1993 at 763 m, **B**) Feb/2019 at 763 m, **C**) Feb/1993 at 902 m, **D**) Feb/2019 at 902 m, **E**) Feb/1993 at 1062 m, and **F**) Feb/2019 at 1062 m.

Figure 9 shows the monthly mean distribution of the theoretical maximum efficiency of a Carnot cycle in the waters around Cuba between 763 and 1062 m, in the months of February/1993 and February/2019. At 736 m depth the thermal efficiency ranged between 0.59 and 0.76, while at depths of 902 and 1062 m, it ranged between 0.68 – 0.81 for 902 m and for 1062 it reaches 0.72 – 0.82, reaching up to 0.85 in small regions north of Havana, mainly in Feb/1993 ( Figure 9E) due to having a lower mean potential temperature than in Feb/2019 ( Figure 9F). In general, the highest ocean thermal efficiency values were obtained at 1062 m depth, not only because it has a lower potential temperature than the rest of the levels, but also because it has a more stable temperature than the rest of the levels, with MAD <= 0.4°C ( Figure 8d).

## Dataset

This data set can be a reference in future for the implementation of OTEC technology in Cuba. Furthermore, research can be carried out aimed at selecting the best places for the installation of OTEC plants, based on the shortest distances from the coast to the isobaths of 1000, 900 and 800 meters; applying internationally established methodologies that are based on the economic, social and cultural context of the selected places, the frequency of extreme meteorological and geological events, etc.

This research has a high level of relevance to national policies as it corresponds to the strategic tasks defined in the State Plan of Cuba to confront climate change (Tarea Vida) and by the Country Program (2020–2024) prepared by the UNDP and reconciled with the Government of Cuba, in particular with the priority of cooperation related to the promotion of sustainable environmental bases, incorporating effects of climate change in the processes of economic and social development by 2030 with the strengthening of national capacities. It is also relevant to the development policies towards 2030 of the nations of Central America and the Caribbean. In this way, there may be a high interest from the Cuban government and through Cuba's collaboration with the Caribbean, joint collaborations can be designed in this sense.

**Figure 10.**
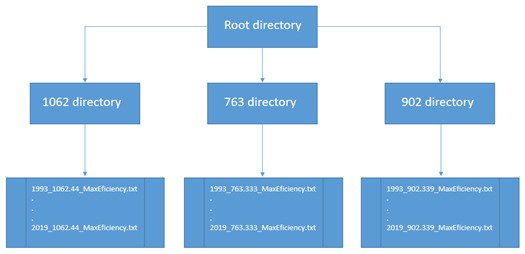
File distribution by directory.

The database for the calculation of thermal efficiency in the seas around Cuba is the result of an investigation conducted by 1. As shown in Figure 10, the dataset includes three folders that contain annual information on thermal efficiency from 1993 to 2019:

(i)  27 files calculated for the 1062m depth level.

(ii)  27 files, calculated for a depth level of 763m, and

(iii)  27 files calculated for the 902m depth level.

Each of the 81 files is stored in comma-separated txt format and 162.68 MB in size.

The same nodes of the regular data mesh of the temperature files **global-reanalysis-phy-001-030-daily** ^1^ for the study area were downloaded from the Copernicus site, and the number of nodes was 145 × 67. A description of the fields of each file separated by commas that do not have a header is presented in Table 2.

**Table 2.** Description of the fields of the output files of the calculation of thermal efficiency. *When it is not possible to calculate the thermal efficiency, either because the point is on land or because the depth level does not exist, the value is -32767.

Description of field	Data type
Date	Date
Level	float
Longitude	float
Latitude	float
Thermal efficiency	float *

## Ethics and consent

Ethics and consent were not required for this study

## Data Availability

The data is available on the Science Data Bank site. The site is free to download and use data sets respecting the corresponding data license. Repository name: Science Data Bank. Dataset name: Thermal Efficiency Dataset Around Cuban Seas (TEDACS). Dataset location: https://www.scidb.cn/en/detail?dataSetId=c36d48ae4d5444e69458e9c80fea84dc DOI: https://doi.org/10.57760/sciencedb.10037 Scientific Data Confirmation Certificate: https://cert.scichain.cn/scidb/2023/08/11/1078085787.en.v1.pdf Publication date: 2023-10-08 This project contains the following data: -  *_1062.44_MaxEficiency.txt -  *_763.333_MaxEficiency.txt -  *_902.339_MaxEficiency.txt *Years: 1993, 1994, 1995 … 2019 Data are made available under the terms of the Creative Commons Attribution 4.0 International License (CC-BY 4.0).
