# Supplementary material for: Thermal Efficiency Dataset for OTEC Applications in Cuban Waters
Source: Open Res Eur. 2025 May 8;4:15. Originally published 2024 Jan 10. [Version 3] doi: 10.12688/openreseurope.16815.3 (PMC11325129; doi:10.12688/openreseurope.16815.3)
Supplement: Peer review history

# Reviewer report: Reviewer response for version 3

**DOI:** 10.21956/openreseurope.21975.r53997

**Published:** 2025-05-27

**Authors:** Erfan Amini

**Affiliations:** 1 Department of Civil, Environmental, and Ocean Engineering, Stevens Institute of Technology (Ringgold ID: 33694), Hoboken, New Jersey, USA

The authors have addressed all the previous comments, and I am in favor of accepting the manuscript for indexing.

Are sufficient details of methods and materials provided to allow replication by others?

Partly

Is the rationale for creating the dataset(s) clearly described?

Yes

Are the datasets clearly presented in a useable and accessible format?

Partly

Are the protocols appropriate and is the work technically sound?

Yes

Reviewer Expertise:

Coastal Engineering, Ocean Energy

I confirm that I have read this submission and believe that I have an appropriate level of expertise to confirm that it is of an acceptable scientific standard.

# Reviewer report: Reviewer response for version 3

**DOI:** 10.21956/openreseurope.21975.r53999

**Published:** 2025-05-16

**Authors:** Dodi Sofyan Arief, Abdul Khair Junaidi

**Affiliations:** 1 Universitas Riau, Pekanbaru,, Indonesia; 2 Mechanical Engineering, Technology College of Pekanbaru, Pekanbaru, Riau, Indonesia

This revised article makes a valuable contribution to the exploration of renewable energy potential in Cuba, specifically focusing on ocean thermal energy. The use of long-term data from the Copernicus Marine Environmental Monitoring Service to calculate Carnot thermal efficiency at various depths demonstrates a strong analytical approach. The revisions—such as the improved title, updated dataset in Kelvin, enhanced geographic detail in the figures, and the inclusion of previously omitted references—significantly strengthen the clarity, accuracy, and overall quality of the manuscript.

Are sufficient details of methods and materials provided to allow replication by others?

Yes

Is the rationale for creating the dataset(s) clearly described?

Yes

Are the datasets clearly presented in a useable and accessible format?

Yes

Are the protocols appropriate and is the work technically sound?

Yes

Reviewer Expertise:

NA

We confirm that we have read this submission and believe that we have an appropriate level of expertise to confirm that it is of an acceptable scientific standard.

# Reviewer report: Reviewer response for version 2

**DOI:** 10.21956/openreseurope.20765.r48872

**Published:** 2024-12-28

**Authors:** Ji Zhang

**Affiliations:** 1 Ocean University of China, Qingdao, China

In my first review, I pointed out that the author’s calculation of the thermal efficiency for OTEC was incorrect. However, it is regrettable that the author has still not corrected this in the revised manuscript. They continue to use Celsius temperatures for the calculation of thermal efficiency, which is unacceptable in thermodynamics, and this mistake is also rudimentary. Although the authors noted that they did not consider the degradation of thermal efficiency in engineering, that is not the issue here. The theoretical thermal efficiency, or the limit thermal efficiency, should be calculated using Kelvin temperatures. Therefore, I suggest that the authors seek out several more authoritative papers on OTEC to truly understand the calculation of OTEC’s thermal efficiency. The Carnot cycle efficiency (the perfect thermodynamic cycle in theory) is approximately 6.7% when the temperature difference is 20 °C. （ Review of enhancement for ocean thermal energy conversion system. In: Journal of Ocean Engineering and Science. Elsevier BV, 2023 [Ref-1]; 8(5): 533–545）

Are sufficient details of methods and materials provided to allow replication by others?

Partly

Is the rationale for creating the dataset(s) clearly described?

Partly

Are the datasets clearly presented in a useable and accessible format?

Yes

Are the protocols appropriate and is the work technically sound?

Partly

Reviewer Expertise:

Ocean thermal energy conversion; energy system

I confirm that I have read this submission and believe that I have an appropriate level of expertise to state that I do not consider it to be of an acceptable scientific standard, for reasons outlined above.

## References

[1] : Review of enhancement for ocean thermal energy conversion system. *Journal of Ocean Engineering and Science* .2023;8(5) : 10.1016/j.joes.2022.03.008 533-545 10.1016/j.joes.2022.03.008

# Response

**Published:** 2024-12-28

**Authors:** Humberto L. Varona

**Affiliations:** Department of Oceanography, Federal University of Pernambuco, Recife, Pernambuco, Brazil

The difference between the Kelvin and Celsius scales lies in the initial reference point. The Kelvin scale is defined from absolute zero, making it an essential absolute scale for scientific computations. In contrast, the Celsius scale, based on the freezing and boiling points of water under normal pressure, is more convenient for everyday applications and practical situations where a relative reference is sufficient. This shift between the two scales, of a constant 273.15 °C, does not affect the temperature differences between specific points, since both scales share the same magnitude of increase per unit. Both scales, therefore, measure the same physical property, but the Kelvin scale provides an indispensable absolute framework in thermodynamics. The mathematical relationship between the scales ensures that, if converted correctly, temperature differences remain invariant, guaranteeing consistency in calculations such as those related to thermal efficiency.  In the context of calculating thermal efficiency, such as in a Carnot cycle, there is no difference in the results when using Celsius or Kelvin, provided that the temperatures are correctly transformed to an absolute scale prior to their application in the equations. This is because thermal efficiency depends on the ratios between absolute temperatures, not on their individual values on a relative scale. The unique scenario in which it would be erroneous to use degrees Celsius directly is if the cold source temperature approaches -273.15 °C, which does not occur under natural conditions because absolute zero is unattainable in practice. In environments such as the ocean, where the lowest observed temperatures are well above absolute zero, such an error would not have any relevance.

# Response

**Published:** 2024-12-29

**Authors:** Humberto L. Varona

**Affiliations:** Department of Oceanography, Federal University of Pernambuco, Recife, Pernambuco, Brazil

On the Celsius temperature scale there is also an under-therm in equation 2 when the cold source temperature is 0°C; this temperature is possible in the ocean due to the presence of salt. The freezing point of seawater ranges from -2.010 to -1.796°C for salinities of 33 to 36.52 psu (Doherty et al., 1974; Fujino et al., 1974; and Kester 1974). This phenomenon is due to cryoscopic lowering, which reduces the freezing point of a solution compared to pure solvent. In our case this did not affect the computation of the thermal efficiency because the minimum temperature in the 3 depth levels used are higher than 0°C. Doherty, Brendan T., and Dana R. Kester. 1974. "Freezing point of seawater." *Journal of Marine Research* 32, (2). https://elischolar.library.yale.edu/journal_of_marine_research/1290 Fujino, K., Lewis, E. L., & Perkin, R. G. (1974). The freezing point of seawater at pressures up to 100 bars. In Journal of Geophysical Research (Vol. 79, Issue 12, pp. 1792–1797). American Geophysical Union (AGU). https://doi.org/10.1029/jc079i012p01792 Kester, D. R. (1974). Comparison of recent seawater freezing point data. In Journal of Geophysical Research (Vol. 79, Issue 30, pp. 4555–4556). American Geophysical Union (AGU). https://doi.org/10.1029/jc079i030p04555 On the other hand, the difference in results when using Celsius versus Kelvin temperatures arises because the ratio between temperatures changes when using a scale with a constant offset such as the Celsius scale. In thermodynamics, temperature ratios must be calculated in terms of their absolute value (Kelvin) because they determine relative energy ratios. The Celsius scale does not reflect the actual ratios because of its 273.15°C offset. Using degrees Celsius ignores that the temperature 0°C is not a minimum energy point; on the contrary 0 K is the correct thermodynamic reference point. **Conclusion:** ***Let's create a new thermal efficiency dataset from the Kelvin temperatures at the hot and cold sources.***

# Response

**Published:** 2024-12-29

**Authors:** Alejandro Rodriguez

**Affiliations:** Center for Marine Meteorology, Institute of Meteorology of Cuba, La Habana, La Habana, Cuba

Dear Ji Zhang.

 We appreciate your review of our work. You are certainly right in your view that the thermal efficiency should be calculated in Kelvin.

 In your first review, we focused on the equations for calculating the thermal efficiency and did not realize that we had to update our dataset by doing the calculations with the temperature values ​​in Kelvin. Please excuse us for such a serious omission.

 We will immediately update our dataset and submit version 3 of our datanote with the mapping of the thermal efficiency around Cuba in Kelvin, as it should be done.

 We strongly request the editors and all reviewers to allow us to submit version 3 of our manuscript with the correct calculation of the thermal efficiency.

 Thank you very much.

# Response

**Published:** 2024-12-30

**Authors:** Alejandro Rodriguez

**Affiliations:** Center for Marine Meteorology, Institute of Meteorology of Cuba, La Habana, La Habana, Cuba

Dear Mr. Ji Zhang.

 We appreciate your review of our manuscript and agree with your rating. It is truly essential to calculate the thermal efficiency in Kelvin because of all the physical implications regarding energy taking the temperature in degrees Celsius, and the indeterminacy of the equation for the value of 0 degrees Celsius.

 We apologize for having overlooked this criterion that you pointed out to us from the first review, we are only focused on improving the equations.

 We will immediately update the dataset and submit version 3 of our datanote.

 Thank you very much.

# Reviewer report: Reviewer response for version 1

**DOI:** 10.21956/openreseurope.18168.r39702

**Published:** 2024-08-14

**Authors:** Ji Zhang

**Affiliations:** 1 Ocean University of China, Qingdao, China

This article provides a detailed analysis of the potential for thermal energy from sea temperature gradients around Cuba, utilizing 27 years of daily data to calculate thermal efficiency at different depths. This long-term data analysis is significant for understanding the availability and variability of thermal energy resources. The study generates 81 files, meticulously recording dates, depths, coordinates, and thermal efficiencies, providing a valuable foundation for future energy planning and environmental impact assessments. Additionally, the research explores applications beyond electricity generation, such as desalination, refrigeration, and aquaculture, highlighting the versatility of thermal energy resources.

 However, it must be noted that the calculation of thermal efficiency presented in the article is wrong. In reality, the maximum thermal efficiency of using sea temperature gradients, even under ideal conditions, is limited by the Carnot cycle efficiency and could not possibly reach around 80%. The author needs to reinforce their understanding of thermodynamics, particularly when dealing with temperature units. Kelvin and Celsius are two different temperature scales, differing in their reference points (zero points) and temperature intervals. The thermal efficiency values reported in the article require further validation and explanation to ensure the accuracy and reliability of the findings.

Are sufficient details of methods and materials provided to allow replication by others?

Partly

Is the rationale for creating the dataset(s) clearly described?

Partly

Are the datasets clearly presented in a useable and accessible format?

Yes

Are the protocols appropriate and is the work technically sound?

Partly

Reviewer Expertise:

Ocean thermal energy conversion; energy system

I confirm that I have read this submission and believe that I have an appropriate level of expertise to confirm that it is of an acceptable scientific standard, however I have significant reservations, as outlined above.

# Response

**Published:** 2024-08-14

**Authors:** Alejandro Rodriguez

**Affiliations:** Center for Marine Meteorology, Institute of Meteorology of Cuba, La Habana, La Habana, Cuba

Dear Ji Zhang

 On behalf of the authors of this paper, I thank you for taking part of your valuable time to review our article.

 Regarding your remarks, I must say that the authors did not consider developing in its entirety the theory of energy transformations in the OTEC generation process. We based ourselves fundamentally on a work published by Adrian Rizki Sinuhaji (https://doi.org/10.18502/ken.v1i1.330), which makes an excellent exposition of the subject; and which arrives, after transformations of the different equations, to an equation that is the one we use for this exercise. This excellent work has been cited by us.

 Obviously, we know that the values ​​of maximum thermal efficiency decrease in practice when the different components of an OTEC plant are installed; but this is a matter for the engineers who are dedicated to building the generating plant. Our work is the calculation of the energy potential that exists in the sea around Cuba, knowing that with a minimum maximum thermal efficiency of 0.7 (this is the conclusion that Sinuhaji reached in his research) it may be feasible to build an OTEC plant in the place, that is, to have a performance of 0.25 to 0.3 as it is known.

 Thank you very much.

 Alejandro Rodríguez

# Response

**Published:** 2024-12-13

**Authors:** Humberto L. Varona

**Affiliations:** Department of Oceanography, Federal University of Pernambuco, Recife, Pernambuco, Brazil

Dear Dr. Ji Zhang Thank you very much for your review and pointing out to improve this research.  Our thermal efficiency computations are correct. As thermal efficiency depends only on the accuracy of hot and cold temperatures, we have added to our research an evaluation of the vertical temperature by comparing the dataset used for the computation with a dataset created from observed data, 6 random points were chosen around the island of Cuba, the bias in all points has been small, mainly at depths 763, 902, and 1062 m. In addition, studies of the spatial and temporal behavior of the Median Absolute Deviation in all the levels used were added, indicating that the spatial and temporal distribution of the temperature in the deep levels is very stable, being this favorable to obtain values of good thermal efficiency. In the first version we already had an evaluation of the surface temperature, comparing this with two sources of observed data, in addition to a study of the temperature anomalies, which are quite variable in the Caribbean Sea, existing regions with remarkable maximum anomalies, this produces a greater temperature gradient between the surface and the deep levels used in the computation of the thermal efficiency, this also contributes to an increase of the efficiency in the Carnot cycle.

# Reviewer report: Reviewer response for version 1

**DOI:** 10.21956/openreseurope.18168.r39698

**Published:** 2024-08-14

**Authors:** Hooi Siang Kang

**Affiliations:** 1 Universiti Teknologi Malaysia, Skudai, Johor, Malaysia

The research presents an exploration of Ocean Thermal Energy Conversion (OTEC) feasibility in Cuba's maritime surroundings, underpinned by bathymetric characterization and thermal efficiency computations. The dataset, compiled over 27 years utilizing Copernicus Marine Environmental Monitoring Service (CMEMS) data, offers insights into thermal efficiency across specific latitudinal and longitudinal bounds.

 The dataset's creation is evidently meticulous based on reliable data sources. The inclusion of CMEMS global ocean reanalysis data ensures a reliable foundation for thermal efficiency calculations. Furthermore, the utilization of MODIS Aqua and WOCE datasets for validation enhances the reliability of the dataset. Protocols employed in the research exhibit its alignment with established practices in OTEC feasibility studies. The computation of thermal efficiency reflects sound scientific methodology. Moreover, the detailed description of methods enhances transparency and facilitates replication by other researchers. The dataset's usability and accessibility are comprehensively documented. Overall, the research provides a potentially valuable contribution to sustainable energy development. 

 The suggested improvements could include conducting sensitivity analyses to assess the impact of various parameters on thermal efficiency calculations. Additionally, detailing quality control measures and the potential impact of SST anomalies on thermal efficiency calculations, would further facilitate research endeavors in utilizing the dataset.

Are sufficient details of methods and materials provided to allow replication by others?

Yes

Is the rationale for creating the dataset(s) clearly described?

Yes

Are the datasets clearly presented in a useable and accessible format?

Yes

Are the protocols appropriate and is the work technically sound?

Yes

Reviewer Expertise:

Ocean Engineering

I confirm that I have read this submission and believe that I have an appropriate level of expertise to confirm that it is of an acceptable scientific standard.

# Response

**Published:** 2024-08-14

**Authors:** Alejandro Rodriguez

**Affiliations:** Center for Marine Meteorology, Institute of Meteorology of Cuba, La Habana, La Habana, Cuba

Dear Hooi Siang Kang

 The authors of this paper thank you for taking part of your valuable time to review our article.

 We are very pleased to receive the satisfactory review from an expert who belongs to a nation that exhibits great strength in the OTEC issue. We are working on version 2 of the paper that we will soon submit for reviewers and the public's consideration.

 Thank you very much.

 Alejandro Rodríguez

# Reviewer report: Reviewer response for version 1

**DOI:** 10.21956/openreseurope.18168.r39145

**Published:** 2024-05-17

**Authors:** Erfan Amini

**Affiliations:** 1 Department of Civil, Environmental, and Ocean Engineering, Stevens Institute of Technology (Ringgold ID: 33694), Hoboken, New Jersey, USA

The authors present a valuable dataset for assessing the thermal efficiency potential in the seas surrounding Cuba, which can contribute to research on Ocean Thermal Energy Conversion (OTEC) technologies and renewable energy exploration in the region. However, there are some limitations and areas that require further clarification or improvement.

 The introduction effectively establishes the context and significance of the research, highlighting the potential of OTEC as a renewable energy source. However, the authors could provide more background information on previous studies or datasets related to thermal efficiency in the region to better situate their work within the existing literature.

 The methods section provides details on the study area, data sources, and the equation used for calculating thermal efficiency. However, there are some limitations and uncertainties associated with the methodology that should be discussed. For example, the authors used reanalysis data with a spatial resolution of 1/12°, which may not capture local variations in thermal efficiency.

 The authors performed data validation using statistical tests to compare the reanalysis data with observed datasets. While this is a useful step, the validation could be more comprehensive. For instance, the authors could compare their thermal efficiency results with any available in-situ measurements or other published datasets in the region to further validate their findings.

 The dataset is well-organized and made publicly available, which is commendable. The figures and tables are informative and support the text. However, some figures, such as the SST trend map (Figure 1) and the spatial distribution of SST (Figure 2), could benefit from a more detailed interpretation and discussion of their implications for thermal efficiency calculations.

 Overall Evaluation: The paper presents a valuable dataset for assessing the thermal efficiency potential in the seas around Cuba. The methodology is generally sound, and the data validation steps enhance the credibility of the results. However, the authors should address the limitations and uncertainties associated with their methodology and dataset more thoroughly. This would help users better understand the potential drawbacks and make informed decisions when using the dataset for their specific applications.

 Suggestions for Improvement:

- Discuss the limitations and uncertainties associated with the spatial and temporal resolution of the reanalysis data and how they may impact the accuracy of the thermal efficiency calculations.
- Provide more background information on previous studies or datasets related to thermal efficiency in the region to better contextualize the current work.
- Expand the data validation by comparing the thermal efficiency results with any available in-situ measurements or other published datasets in the region.
- Provide a more detailed interpretation and discussion of the implications of the SST trend map and spatial distribution of SST for thermal efficiency calculations.
- Discuss the potential limitations and uncertainties associated with the dataset and how they may affect its usability for different applications.

Are sufficient details of methods and materials provided to allow replication by others?

Partly

Is the rationale for creating the dataset(s) clearly described?

Yes

Are the datasets clearly presented in a useable and accessible format?

Partly

Are the protocols appropriate and is the work technically sound?

Yes

Reviewer Expertise:

Ocean Renewable Energy, Desing Optimization, Coastal Infrastructures

I confirm that I have read this submission and believe that I have an appropriate level of expertise to confirm that it is of an acceptable scientific standard, however I have significant reservations, as outlined above.

# Response

**Published:** 2024-05-18

**Authors:** Alejandro Rodriguez

**Affiliations:** Center for Marine Meteorology, Institute of Meteorology of Cuba, La Habana, La Habana, Cuba

Dear Reviewer Erfan Amini,   Thank you very much for your time for reviewing and suggestioning. Comments regarding the limitations of the spatial and temporal resolution of the data used in the research will be added in the next version of the article and mention will be made of previous studies in the area (there are really few). We will also expand the discussion of the implications of the sea surface temperature trend, and work to show the limitations associated with the data set. Thanks a lot.

# Reviewer report: Reviewer response for version 1

**DOI:** 10.21956/openreseurope.18168.r39696

**Published:** 2024-05-16

**Authors:** Dodi Sofyan Arief, Abdul Khair Junaidi

**Affiliations:** 1 Universitas Riau, Pekanbaru,, Indonesia; 2 Mechanical Engineering, Technology College of Pekanbaru, Pekanbaru, Riau, Indonesia

1. How was the process of calculating thermal efficiency conducted in this study?
2. Why were depths of 763, 902, and 1062 meters chosen as the focus of research for exploiting sea thermal energy?
3. What insights can be gained from the presented dataset regarding the potential development of renewable energy in the vicinity of Cuba?
4. What is the data quality of the Copernicus Marine Environmental Monitoring Service (CMEMS) GLOBAL_MULTIYEAR_PHY_001_030 product used in this research?
5. What are the potential implications of the thermal efficiency findings from this research on the energy industry and environment in Cuba or surrounding regions?
6. What potential future research directions or areas can be developed based on the findings of this research data in the context of OTEC system development?

The literature review is not sufficient for the given title. Try to expand the reviewed references by prioritizing publications. The following papers are just suggestions and can help improve the given discussion:

 (Koto, 2012) (Ref-1)

 (Koto et al.,2017)(Ref-2)

 (Koto et al.,2016)(Ref-3)

 (Koto et al.,2012)(Ref-4)

Are sufficient details of methods and materials provided to allow replication by others?

Yes

Is the rationale for creating the dataset(s) clearly described?

Yes

Are the datasets clearly presented in a useable and accessible format?

Yes

Are the protocols appropriate and is the work technically sound?

Yes

Reviewer Expertise:

Ocean thermal energy conversion (OTEC) is a renewable energy technology (Mechanical Engineering)

We confirm that we have read this submission and believe that we have an appropriate level of expertise to confirm that it is of an acceptable scientific standard, however we have significant reservations, as outlined above.

## References

[1] : Potential of Ocean Thermal Energy Conversion in Indonesia. *https://isomase.org/IJERCE/Vol.4%20Oct%202016/4-1.pdf* .

[2] : Sea Temperatures Profiles for Ocean Thermal Energy Conversion in Siberut-Mentawai, Sumatera Barat, Indonesia. *https://isomase.org/JSOse/Vol.11%20Sep%202017/11-2.pdf* .

[3] : Preliminary Study on Ocean Thermal Energy Conversion in Siberut Island, West Sumatera, Indonesia. *https://isomase.org/JAse/Vol.6%20May%202016/6-1.pdf* .

[4] : Ocean Thermal Energy Conversion in Indonesia, Malaysia and Philippines. *https://isomase.org/Journals/index.php/ijerce/article/view/94/70* .

# Response

**Published:** 2024-05-18

**Authors:** Alejandro Rodriguez

**Affiliations:** Center for Marine Meteorology, Institute of Meteorology of Cuba, La Habana, La Habana, Cuba

Dear Dodi Sofyan Arief

 We thank you for taking your time to review our article.

 All your comments, suggestions and bibliographic references suggested by the reviewer will be taken into account in the next version of the article.

 We will take all the questions you have asked into account for version 2 of the manuscript. We truly believe that it will raise its scientific level. We will study and use, in the new version, the suggested bibliography.

 Thank you so much.

# Response

**Published:** 2025-05-09

**Authors:** Dodi Sofyan Arief

**Affiliations:** Mechanical Engineering, Riau University, Pekanbaru, Riau, Indonesia

Dear Alejandro Rodriguez, Thank you for considering the feedback and suggestions I provided on your article. I am pleased to know that the comments and bibliographic references I suggested were found to be helpful. It is encouraging that you plan to incorporate them into the next version of your manuscript. Addressing these points will certainly enhance the scientific quality of your work. Best regards,

 Dodi Sofyan Arief
